# Differentiating Pediatric Bipolar Disorder, Attention-Deficit/Hyperactivity Disorder, and Other Psychopathologies Using Self-Reported Mood and Energy Data and Actigraphy Findings: Correlation and Machine Learning–Based Prediction of Mood Severity

**DOI:** 10.2196/78163

**Published:** 2025-12-04

**Authors:** Rasim S Diler, Farzan Vahedifard, Boris Birmaher, Satish Iyengar, Maria Wolfe, Brianna N Lepore, Mariah Chobany, Halimah Abdul-Waalee, Greeshma Malgireddy, Jonathan A Hart, Michele A Bertocci

**Affiliations:** 1 Department of Psychiatry, University of Pittsburgh, 3811 O’Hara Street, Pittsburgh, PA, 15213, United States, 1 3125136325

**Keywords:** bipolar disorder, ADHD, attention-deficit/hyperactivity disorder, actigraphy, mood, energy, machine learning, adolescents

## Abstract

**Background:**

Distinguishing pediatric bipolar disorder (BD) from attention-deficit/hyperactivity disorder (ADHD) is challenging due to overlapping fluctuations in mood, energy, and activity. Combining objective actigraphy with self-reported mood and energy data may aid differential diagnosis and risk monitoring.

**Objective:**

This study aimed to test same-day associations between actigraphy-derived activity extremes and self-reported mood and energy, and to evaluate whether these measures predict same-day and next-day severe mood in adolescents with BD, ADHD, and other diagnoses.

**Methods:**

We analyzed 209 inpatients (2148 patient-days) across 4 groups (ADHD without BD: n=54; BD with ADHD: n=42; BD without ADHD: n=34; other diagnoses: n=79). Actigraphy data (Philips Actiwatch 2) were summarized into daily maximum and minimum quartiles (Max1-Max4 and Min1-Min4). Mood and Energy Thermometer (−10 to +10) ratings were categorized as follows: OK (<3), mild (3‐4), moderate (5‐6), and severe (>6). Group differences used Kruskal-Wallis and Mann-Whitney *U* tests with Bonferroni correction (*P*<.004). Associations used chi-square tests with Cramér V. Leak-safe machine learning (patient-wise GroupKFold) classified SevereDay (same day) and SevereTomorrow (next day) using actigraphy, sleep, energy, and demographic data.

**Results:**

BD without ADHD showed the tightest coupling of extreme activity with negative mood and energy (Cramér V of up to 0.24; *P*<.004). ADHD without BD showed stronger links between activity and positive energy. Machine learning achieved a receiver operating characteristic area under the curve (ROC-AUC) of 0.85, an accuracy of 0.79, and an *F*_1_-score of 0.67 for SevereDay. SevereTomorrow performance was moderate (ROC-AUC=0.80; accuracy=0.79; *F*_1_-score=0.60). Energy variability and actigraphy averages/peaks were the top predictors.

**Conclusions:**

Integrating actigraphy, sleep, and daily energy ratings identifies severe mood days and provides early next-day risk signals in hospitalized adolescents. The findings support wearable-based phenotyping for precision monitoring, with external validation needed in outpatients.

## Introduction

The early and accurate diagnosis of bipolar disorder (BD) in youth is critical, given the high risk of both over- and underdiagnosis in clinical practice. On average, it takes nearly a decade to distinguish mania or hypomania from other overlapping clinical presentations, contributing to delays in treatment initiation [1,2]. BD and attention-deficit/hyperactivity disorder (ADHD) in youth pose significant challenges for psychiatric assessment due to marked variability in mood, energy, attention, and activity levels [3,4]. Traditional psychiatric evaluations rely heavily on retrospective self-reports and caregiver reports, which are prone to recall errors and informant disagreement. Clinicians also have limited time to establish mood timelines, making it difficult to capture the episodic nature of BD. Actigraphy provides an objective measure of activity that can detect fluctuations characteristic of BD and ADHD [5,6]. However, unlike well-established physiological indices, such as heart rate, blood pressure, and blood glucose, actigraphy-derived activity counts (ACs) lack standardized clinical interpretation. Prior work has primarily examined mean ACs, but extreme values (maximum and minimum) and the overall range may better capture mood-energy fluctuations. These metrics could offer individualized, clinically relevant insights into the dynamic nature of these disorders and their links to subjective self-reports [7,8].

The Mood and Energy Thermometer (MET) has been developed to standardize patient-family-clinician communication by rating low mood and low energy on a scale of 10 to 0 and elated mood and high energy on a scale of 0 to +10. Incorporating energy ratings alongside mood ratings may be especially valuable, as energy levels are often observable to others and less subject to interpretive bias than mood alone. Integrating objective actigraphy data with subjective or semisubjective mood and energy assessments may provide a more comprehensive picture of pediatric psychiatric presentations and help identify BD earlier and more reliably [9].

Incorporating energy levels into the classification of mood disorders aligns closely with the DSM-5 (Diagnostic and Statistical Manual of Mental Disorders, Fifth Edition) classification system, where energy level is a primary mood symptom criterion. The DSM-5 explicitly states that for a diagnosis of bipolar mania, there must be an increase in energy or activity in addition to mood changes, marking a significant shift from previous editions where mood changes alone sufficed for diagnosis [10,11]. This change underscores the importance of energy levels as a critical component in understanding mood disorders, particularly in BD, where the presence of increased energy is essential for diagnosing manic episodes [11]

This study aimed to improve diagnostic precision in BD and ADHD by examining associations between self-reported mood and energy states (via the MET) and extreme actigraphy-derived activity metrics in adolescents hospitalized for mood and behavioral disorders. Participants were categorized into 4 diagnostic groups: “BD without ADHD,” “BD with ADHD,” “ADHD without BD,” and “other diagnoses” (eg, depression and disruptive behavior disorders). Misdiagnosis—especially confusion between “BD with ADHD” comorbidity and “ADHD without BD”—can delay appropriate treatment and worsen outcomes. We hypothesized that actigraphy-based indices of extreme activity levels (peaks, troughs, and ranges), when combined with self-reported mood and energy ratings, would reveal distinct group-specific patterns and help differentiate BD from ADHD and other conditions.

In addition to traditional statistical analyses, we implemented machine learning methods to explore whether actigraphy- and energy-derived features could predict daily mood severity. Beyond modeling continuous mood means, we evaluated whether integrated actigraphy and energy features could (1) classify clinically severe mood days and (2) forecast next-day severity. Participants were recruited from the Inpatient Child and Adolescent Bipolar Services unit at Western Psychiatric Hospital, where diagnoses were confirmed through structured assessments and consensus conferences under the leadership of the first author (RSD), a board-certified child psychiatrist specializing in pediatric BD. This inpatient setting provided a unique opportunity to monitor dynamic mood-energy-activity interactions in real-world clinical care.

## Methods

### Ethical Considerations

This study was approved by the Human Research Protection Office at the University of Pittsburgh Institutional Review Board (ID STUDY21110097). Data were collected as part of clinical care for mood monitoring during inpatient stay and not as part of a funded research study, and thus, participants were not compensated for their data. All data were fully deidentified before analysis, and the Institutional Review Board determined that additional informed consent was not required. Patient privacy and confidentiality were maintained throughout the study.

### Study Population

This retrospective study analyzed data from 459 adolescent inpatients at the Western Psychiatric Hospital, University of Pittsburgh Medical Center, collected from 2014 to 2023. Participants, aged 13 to 18 years, were diagnosed with well-characterized BD without ADHD (mean age 14.97, SD 1.36 years; 82% [28/34] female), BD with ADHD (mean age 15.33, SD 1.91 years; 57% [24/42] female), ADHD without BD (mean age 15.00, SD 1.39 years; 44% [24/54] female), and other diagnoses (mean age 15.23, SD 1.46 years; 75% [59/79] female). The other diagnoses group included patients with a variety of psychiatric disorders (anxiety disorders: generalized anxiety, panic disorder, social anxiety, social phobia, separation anxiety disorder, and other anxiety disorders), disruptive behavior disorders (conduct disorder, oppositional defiant disorder, and intermittent explosive disorder), obsessive-compulsive and related disorders, substance use disorders, and trauma and related disorders (posttraumatic distress disorder, acute stress disorder, and adjustment disorder), each potentially displaying different activity levels. Since our focus was on BD, we made sure that our participants in the other diagnoses group, including those with major depressive disorder, had no history of manic episodes (based on DSM-5 criteria and expert review) to avoid any overlap with BD. The study included all inpatients at the Western Psychiatric Hospital who completed MET daily ratings and wore an actigraphy device during their hospitalization while they were receiving diagnostic assessments and treatments. The study excluded patients with a diagnosis of autism spectrum disorder (levels II and III), intellectual disability, eating disorder, schizophrenia, or severe substance use disorder. After processing and cleaning of actigraphy data according to previously published studies (eg, standardized visual editing procedures and inclusion of additional semiautomated quality assurance procedures on Actiware [Phillips Resipronics], including identification of the main rest interval [defined as the longest rest interval each day] and removal of invalid sleep intervals containing ≥1 hour of off-wrist time or recording errors) [12,13], 209 participants were included in the analysis.

### Clinical Assessment

RSD, a licensed, board-certified pediatric psychiatrist specializing in pediatric BD and the director of the Inpatient Child and Adolescent Bipolar Services unit at Western Psychiatric Hospital, directly trained all clinical diagnostic staff and confirmed all psychiatric diagnoses through consensus meetings. The DSM-5 criteria were used for the final identification of BD-I/II, and other specified bipolar was defined as the presence of brief (4 hours/day and 4 in a lifetime) cluster DSM manic symptoms that caused impairment. All instruments had good psychometric properties and included the Kiddie Schedule for Affective Disorders and Schizophrenia for School-Age Children (K-SADS) [14].

### MET Assessment

The self-reported MET, developed at the Western Psychiatric Hospital, rates mania and increased energy on a scale of 1 to 10 points and depression and tiredness on a scale of −1 to −10 points [1]. Each participant completed the MET scale each morning and evening, and separate scores for mood and energy were averaged across the day. For this study, we included (1) the highest and lowest scores for elated mood (“MoodPosMax” and “MoodMin”) and elated energy (“EnergyPosMax” and “EnergyMin”) on the MET (scale from 0 to +10 points), (2) the highest and lowest scores for depressed mood (“MoodNegMax” and “MoodNegMin”) and depressed energy (“EnergyNegMax” and “EnergyNegMin”) on the MET (scale from 10 to 0 points), and (3) the range for mood (“MoodRange”) and energy (“EnergyRange”) on the MET (scale from −10 to +10 points).

Patients with BD (feeling ok, experiencing mania, or experiencing depression) assessed their mood and energy levels daily using this scale. Our master’s degree clinician (MW) met with the patients daily to assist in identifying and recording mood symptoms.

### Actigraphy Monitoring

Continuous activity monitoring was conducted using the Philips Actiwatch 2 (Phillips Respironics), worn on the nondominant wrist, which recorded physical activity in 1-minute epochs throughout each patient’s hospitalization, capturing both daytime activity and sleep patterns. To ensure a robust activity profile aligned with clinical diagnostic criteria for BD, which requires a minimum of 4 days of observation, only patients with at least 4 consecutive days of actigraphy data were included. The initial dataset comprised 459 adolescent inpatients admitted between 2014 and 2023, and it underwent rigorous quality control: 6 cases were excluded due to missing demographic data, 29 cases were removed for insufficient actigraphy data (eg, admission or discharge days or fewer than 4 days of recording), and 35 additional cases were excluded for incomplete mood or energy scores, resulting in 389 cases. Further refinement excluded 180 cases with zero values for all mood or energy reports on over 70% of days in order to focus on clinically informative data, yielding a final cohort of 209 adolescents (2148 patient-days). This study involved a diverse cohort, with the highest participation from the “other diagnoses” group (79 unique IDs and 799 days recorded). This was followed by the “ADHD without BD” group (54 unique IDs and 572 days), “BD with ADHD” group (42 unique IDs and 444 days), and “BD without ADHD” group (34 unique IDs and 333 days).

To quantify activity extremes, a custom Python algorithm (*Automatic Max and Min Identifier*) identified 4 nonoverlapping 60-minute maximum (Max1st-Max4th) and minimum (Min1st-Min4th) activity periods daily between 07:00 AM and 09:59 PM. Differences between maxima and minima (eg, Max4 – Min4) were calculated to assess activity variability, and timestamps for peak and trough periods were recorded to evaluate diurnal patterns. Continuous actigraphy data were categorized into quartiles (Max1st-Max4th and Min1st-Min4th) using Python’s pandas.qcut() function, ensuring equal observation distribution to mitigate skewness. Mood and energy scores from the MET were classified into severity ranges: *OK* (<3), *mild* (3-4), *moderate* (5-6), and *severe* (>6), aligning with clinical standards.

### Statistical Analysis

We used nonparametric tests to evaluate differences in mood and energy variables across diagnostic groups. Kruskal-Wallis tests assessed overall group differences, and pairwise Mann-Whitney *U* tests with Bonferroni correction controlled for multiple comparisons (adjusted significance threshold: *P*<.004). Associations between actigraphy-derived activity quartiles (Max1-Max4 and Min1-Min4) and mood and energy severity categories from the MET were examined using chi-square tests with Cramér V effect sizes. Effect sizes were interpreted using established conventions: negligible (0‐0.1), weak (0.1‐0.2), moderate (0.2‐0.3), and strong (>0.3). All data were anonymized to ensure confidentiality.

### Machine Learning Modeling of Daily Mood Severity

#### Prediction Targets

Two binary outcomes were defined using mood-only labels from the MET to avoid target leakage from energy: (1) *SevereDay* (same day), labeled severe if MoodPosMax ≥7 or MoodNegMax ≥7; and (2) *SevereTomorrow* (next day), defined by shifting the same rule forward 1 day within each patient. Energy variables were included only as predictors and not in the labels.

#### Features

Candidate predictors spanned four domains:

1. Actigraphy: daily peak and trough windows and variability (Max1st-Max4th, Min1st-Min4th, and Max-Min contrasts), total AC, average AC/minute, average AC/epoch, standard AC, and maximum AC
2. Sleep: duration, efficiency, wake after sleep onset, sleep time, percentage sleep, and fragmentation
3. Self-reported energy: EnergyPosMax, EnergyPosMin, EnergyNegMax, EnergyNegMin, EnergyMin, EnergyMax, EnergyMean, and EnergyRange
4. Demographics: Age in months and categorical variables (eg, gender and diagnostic label)

#### Preprocessing and Leakage Control

All preprocessing steps (median imputation for numeric features, one-hot encoding for categorical variables, and scaling where required) were applied within the pipeline and fit only on training folds. Class imbalance was addressed with the Synthetic Minority Oversampling Technique (SMOTE) applied only to training data. Group identity (patient ID) was used in all splits to prevent cross-patient leakage.

#### Validation Design

Model generalization was estimated with 5-fold GroupKFold cross-validation. Hyperparameters were tuned using inner GroupKFold (3-fold) with receiver operating characteristic area under the curve (ROC-AUC) as the selection metric. Decision thresholds were optimized within each training fold to balance accuracy and *F*_1_-score.

#### Models Compared

Logistic regression, histogram-based gradient boosting (HistGradientBoosting), random forest, extra trees, and extreme gradient boosting (XGBoost; when available) were evaluated, along with a soft-vote ensemble.

#### Evaluation Metrics

Performance was assessed using ROC-AUC, precision-recall area under the curve (PR-AUC), accuracy, and *F*_1_-score averaged across folds. For the best-performing model in the final fold, we report confusion matrices, ROC/PR curves, and threshold calibration plots.

## Results

### Sample Characteristics and Group Sizes

Statistical analyses were conducted in three phases: (1) group comparisons of mood and energy variables using Kruskal-Wallis tests and pairwise Mann-Whitney *U* tests with Bonferroni correction (*P*_corrected_<.004); (2) associations between categorized actigraphy-based activity levels (quartiles) and mood/energy severity ranges using chi-square tests with Cramér V effect sizes; and (3) machine learning classification (logistic regression, HistGradientBoosting, random forest, extra trees, and XGBoost) to predict daily mood severity (SevereDay and SevereTomorrow), based on actigraphy-derived features and self-reported energy scores. Feature importance was extracted to identify the top predictors within each diagnostic group.

### Demographic Information and Group Comparisons of Mood and Energy

Table 1 summarizes the demographic and clinical characteristics. The “BD with ADHD” and “other diagnoses” groups included a higher proportion of females than the “ADHD without BD” group. Participants in these 2 groups were also older than those in the “ADHD without BD” and “BD without ADHD” groups.

Kruskal-Wallis tests, corrected for multiple comparisons (*P*_corrected_<.004), revealed significant differences across diagnostic groups for most mood and energy variables (Multimedia Appendix 1). For mood ratings, the “BD without ADHD” and “ADHD without BD” groups reported the highest maximum positive mood scores (MoodPosMax), whereas the “other diagnoses” group showed the highest maximum and minimum negative mood scores (MoodNegMax and MoodNegMin) and the widest mood range. Minimum positive mood (MoodMin) was the lowest in the “BD with ADHD” and “ADHD without BD” groups.

For energy ratings, the “ADHD without BD” and “BD without ADHD” groups showed the highest maximum positive energy (EnergyPosMax), while the “other diagnoses” group exhibited the most severe negative energy states (EnergyNegMax and EnergyNegMin) and the broadest energy range. Minimum positive energy (EnergyPosMin) was the highest in the “ADHD without BD” group and the lowest in the “BD with ADHD” and “BD without ADHD” groups. Overall, the “BD without ADHD” and “ADHD without BD” groups were characterized by elevated positive mood and energy and lower variability, whereas the “other diagnoses” group—largely including depressive disorders—was marked by greater negative affect and instability.

**Table 1.** Demographic information and target mood and energy data across the diagnostic labels.

Variable	ADHD^a^ without BD^b^	BD with ADHD	BD without ADHD	Other diagnoses	Total	H-statistic	*P* value
Demographics							
Number of patients	54	42	34	79	209	23.26	<.001
Number of days	572	444	333	799	2148	30.48	<.001
Female patient-days	323	322	251	543	1439	2147	<.001
Male patient-days	188	102	50	127	467	2147	<.001
Mood/energy variable, mean (SD)							
MoodPosMax	1.71 (3.10)^c^	1.29 (2.70)^d^	1.54 (2.60)^c^	1.26 (2.43)^d^	—^e^	78.19	<.001
MoodNegMax	1.48 (3.11)^f^	1.24 (2.56)^f^	1.62 (2.67)^d^	2.46 (3.59)^c^	—	74.90	<.001
MoodPosMin	0.92 (2.47)	0.72 (2.13)	0.66 (1.91)	0.62 (1.77)	—	89.31	<.001
MoodNegMin	0.67 (2.15)^f^	0.63 (1.90)^f^	0.76 (1.84)^d^	1.76 (3.24)^c^	—	72.92	<.001
MoodMin^g^	−0.62 (4.26)^c^	−0.56 (3.55)^c^	−1.07 (3.48)^d^	−1.9 (4.32)^f^	—	11.54	.009
MoodRange	3.19 (4.25)^d^	2.52 (3.67)^f^	3.16 (3.81)^d^	3.72 (3.88)^c^	—	90.86	<.001
EnergyPosMax	2.02 (3.36)^c^	1.47 (2.91)^d^	1.66 (2.65)^c^	1.38 (2.70)^d^	—	71.50	<.001
EnergyNegMax	1.12 (2.42)^f^	1.25 (2.30)^f^	1.37 (2.20)^d^	2.41 (3.43)^c^	—	117.23	<.001
EnergyPosMin	1.11 (2.72)^c^	0.85 (2.34)^d^	0.65 (1.85)^d^	0.83 (2.23)	—	70.58	<.001
EnergyNegMin^g^	0.46 (1.55)^f^	0.64 (1.65)^d^	0.57 (1.43)^d^	1.71 (3.09)^c^	—	8.97	.03
EnergyMin	−0.03 (3.95)^c^	−0.46 (3.53)^d^	−0.75 (3.14)^d^	−1.62 (4.54)^f^	—	98.16	<.001
EnergyRange^g^	3.13 (4.06)^f^	2.72 (3.60)^f^	3.03 (3.35)^d^	3.79 (3.77)^c^	—	10.80	.01
MoodMean	0.87 (4.74)	−0.07 (4.13)	−1.79 (4.71)	−0.03 (3.81)	—	96.08	<.001
EnergyMean	1.30 (4.79)	0.16 (4.13)	−1.60 (4.77)	0.10 (3.56)	—	117.59	<.001

a ADHD: attention-deficit/hyperactivity disorder.

b BD: bipolar disorder.

c Values with this footnote are significantly higher than values with the “d” and “f” footnotes in the row.

d Values with this footnote are significantly lower than values with the “c” footnote but significantly higher than values with the “f” footnote in the row.

e Not applicable.

f Values with this footnote are significantly lower than values with the “c” and “d” footnotes in the row.

g Variable did not meet the correction significant level (*P*<.004).

### Pairwise Comparisons of Mood and Energy

Pairwise contrasts (Multimedia Appendix 2), adjusted with Bonferroni correction (*P*_corrected_<.004), confirmed distinct symptom profiles across diagnostic groups. The “ADHD without BD” and “BD without ADHD” groups exhibited significantly higher maximum positive mood (MoodPosMax) and positive energy (EnergyPosMax) compared to the “BD with ADHD” and “other diagnoses” groups (*P*<.001). In contrast, the “other diagnoses” group consistently showed the most severe negative mood and energy scores (MoodNegMax, MoodNegMin, EnergyNegMax, and EnergyNegMin; all *P*<.001). Within the BD subtypes, “BD without ADHD” reported higher negative mood than “BD with ADHD” (*P*<.01). Overall, “BD with ADHD” presented the least variability across mood and energy domains.

### Box Plots, Density Plots, and Severity Distributions

Box plot visualizations (Multimedia Appendix 1) highlighted that the “other diagnoses” group experienced disproportionately higher negative mood and energy scores, with several outliers indicating extreme cases.

Kernel density estimates for mood and energy variables across diagnostic groups are shown in Multimedia Appendix 3. Most participants across all groups clustered in the “OK” range (<3), consistent with mild symptoms. However, “ADHD without BD” was overrepresented in the upper ranges of positive mood and energy, while “other diagnoses” was overrepresented in severe negative states. The “ADHD without BD” group had the greatest number of severe cases for positive mood and energy, whereas the “other diagnoses” group contributed the largest share of severe negative mood and energy cases. The “BD without ADHD” group generally had the fewest severe cases, consistent with a comparatively more stable profile.

### Variability Analyses

Additional variability analyses (SD or IQR and distributional summaries) are presented in Multimedia Appendix 4, which revealed that energy variables fluctuated more widely than mood variables across all groups. The “other diagnoses” group displayed the greatest instability, with the largest SDs for negative mood (MoodNegMax SD=3.86) and energy (EnergyNegMax SD=3.65). By comparison, the “ADHD without BD” and “BD with ADHD” groups showed similar variability, while the “BD without ADHD” group exhibited narrower energy ranges (EnergyRange: 17 vs 20 in other groups), suggesting a more constrained but distinct fluctuation pattern. IQR analyses reinforced these findings. The “other diagnoses” group had the widest IQRs for negative mood (MoodNegMax IQR=7) and energy (EnergyNegMax IQR=6), whereas the “BD with ADHD” group showed tighter distributions for positive mood (MoodPosMax IQR=3).

### Correlation of Actigraphy With Mood and Energy

The correlation matrix between activity quartiles and mood and energy variables is shown in Multimedia Appendix 5.

#### Part A: Regression Analyses

**Figure 1.**
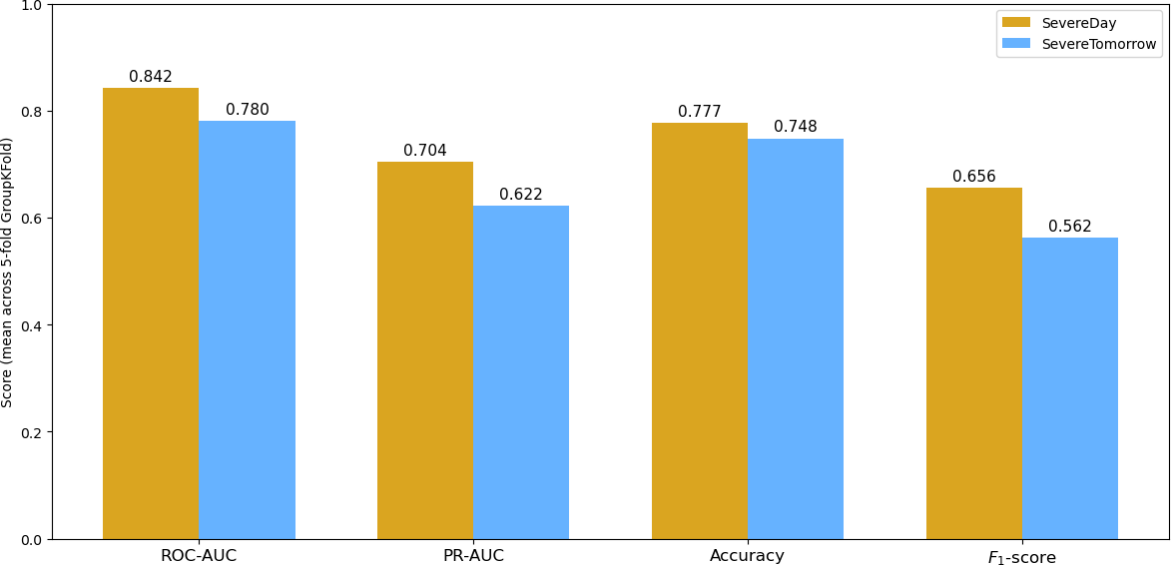
Leak-safe classification performance for same-day and next-day mood prediction. Performance of the best model (logistic regression) in predicting same-day mood severity (SevereDay) and next-day mood severity (SevereTomorrow) using leak-safe, patient-wise cross-validation. Bars show the mean receiver operating characteristic area under the curve (ROC-AUC), precision-recall area under the curve (PR-AUC), accuracy, and *F*_1_-score across 5-fold GroupKFold validation; values are annotated above each bar. The same-day model achieved recall of 0.75 and precision of 0.61, whereas the next-day model achieved recall of 0.54 and precision of 0.67.

Regression models failed to establish meaningful predictive relationships between actigraphy-derived activity variables and mood and energy outcomes. Linear regression yielded a modest mean squared error (16.62) and explained virtually no variance (*R*²=0.015). Decision tree, random forest, and XGBoost models performed worse, with higher mean squared error values (27.06, 18.38, and 20.33, respectively) and negative *R*² scores (−0.59 to −0.23). These findings suggest that actigraphy alone was insufficient to capture the complexity of mood-energy dynamics, likely reflecting the nonlinear and heterogeneous nature of affective fluctuations as well as unmeasured contextual factors such as sleep disturbances and medication effects. Because regression on continuous mood performed poorly and carried risks of target leakage, subsequent artificial intelligence (AI) analyses were reframed using a binary, leak-safe severity framework (Figure 1).

#### Part B: Chi-Square Tests

**Figure 2.**
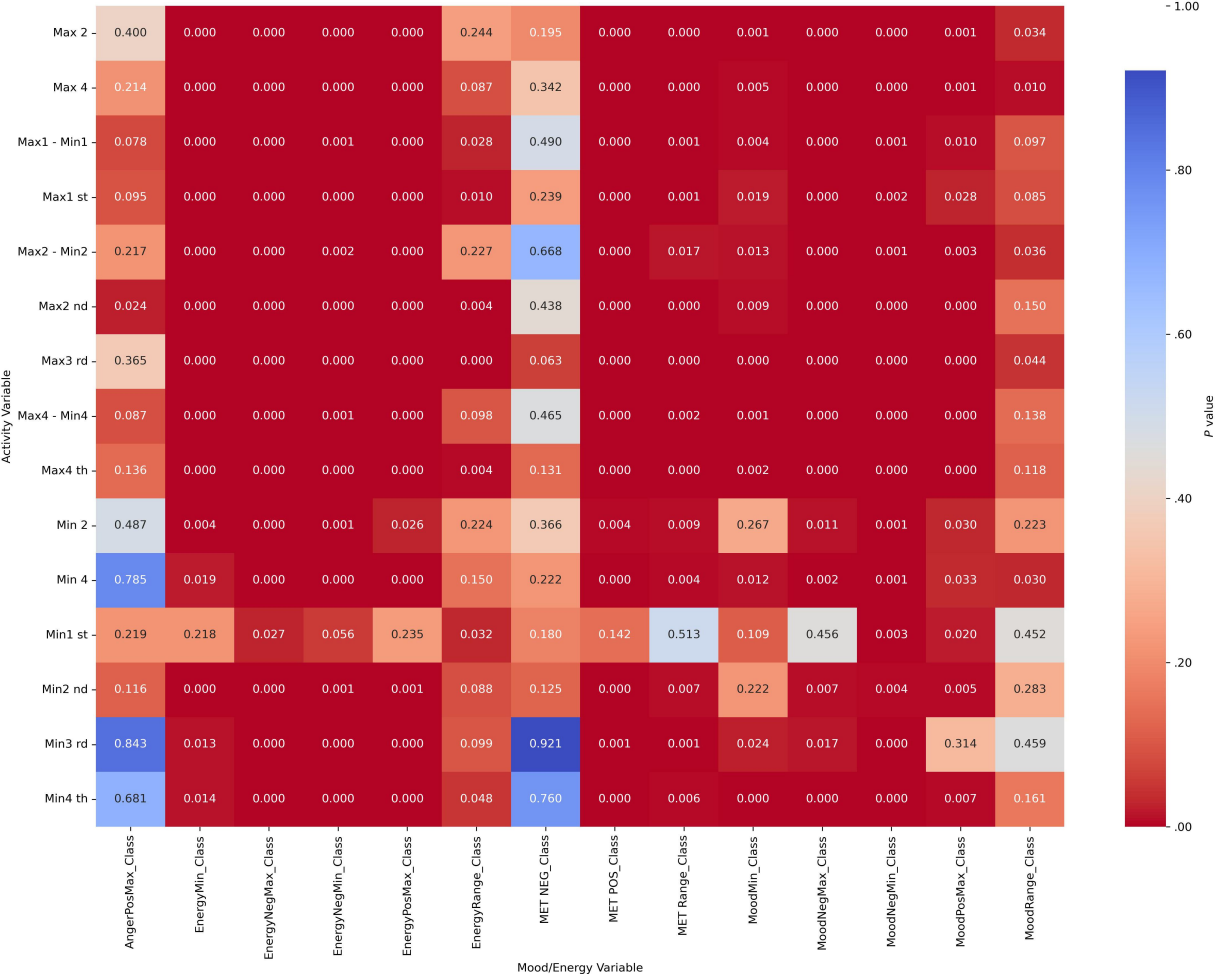
Heatmap visualization to illustrate the *P* values of activity (actigraphy quartiles: Max1-Max4 and Min1-Min4; “Max1…Max4” and “Min1…Min4” refer to quartiles of 4 nonoverlapping 60-min peak/trough windows per day [07:00 AM-09:59 PM]; 1=lowest quartile, 4=highest quartile) and mood/energy category (MoodPosMax/Min, MoodNegMax/Min, EnergyPosMax/Min, EnergyNegMax/Min, and range=daily max-min) correlations for attention-deficit/hyperactivity disorder without bipolar disorder. Max: maximum; MET: Mood and Energy Thermometer; Min: minimum; Neg: negative; Pos: positive.

Chi-square analyses revealed significant associations between categorized actigraphy quartiles and mood and energy severity ranges across diagnostic groups (all *P*_corrected_<.004). These results indicate that mood and energy distributions vary systematically with activity levels. Heatmap visualizations (Figures2^5^) highlight these associations, with darker red cells corresponding to lower *P* values, denoting stronger statistical significance. Importantly, while the chi-square test confirmed significant associations, it did not quantify the strength of the effect.

**Figure 3.**
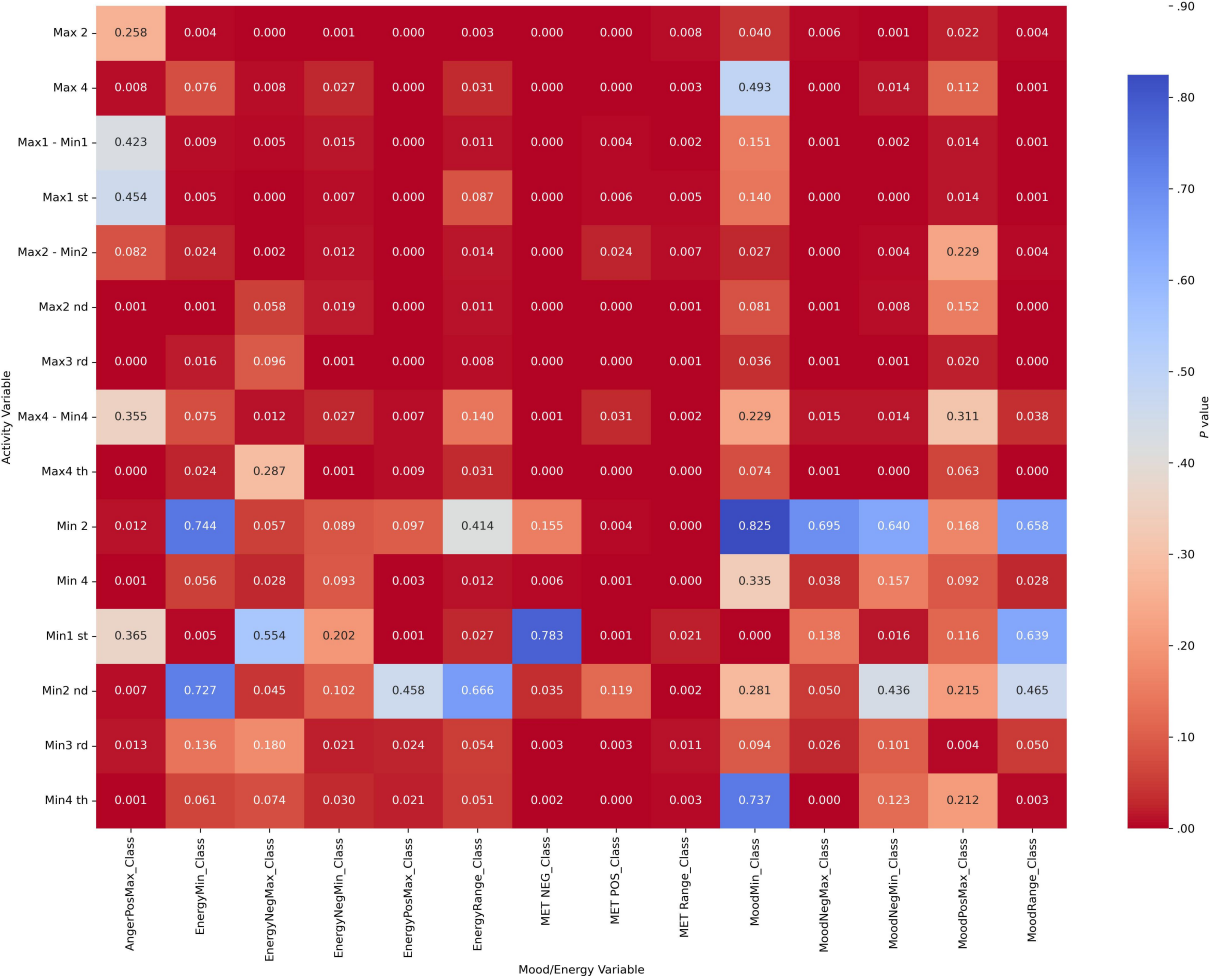
Heatmap visualization to illustrate the *P* values of activity (actigraphy quartiles: Max1-Max4 and Min1-Min4; “Max1…Max4” and “Min1…Min4” refer to quartiles of 4 nonoverlapping 60-min peak/trough windows per day [07:00 AM-09:59 PM]; 1=lowest quartile, 4=highest quartile) and mood/energy category (MoodPosMax/Min, MoodNegMax/Min, EnergyPosMax/Min, EnergyNegMax/Min, and range=daily max-min) correlations for bipolar disorder with attention-deficit/hyperactivity disorder*.* Max: maximum; MET: Mood and Energy Thermometer; Min: minimum; Neg: negative; Pos: positive.

**Figure 4.**
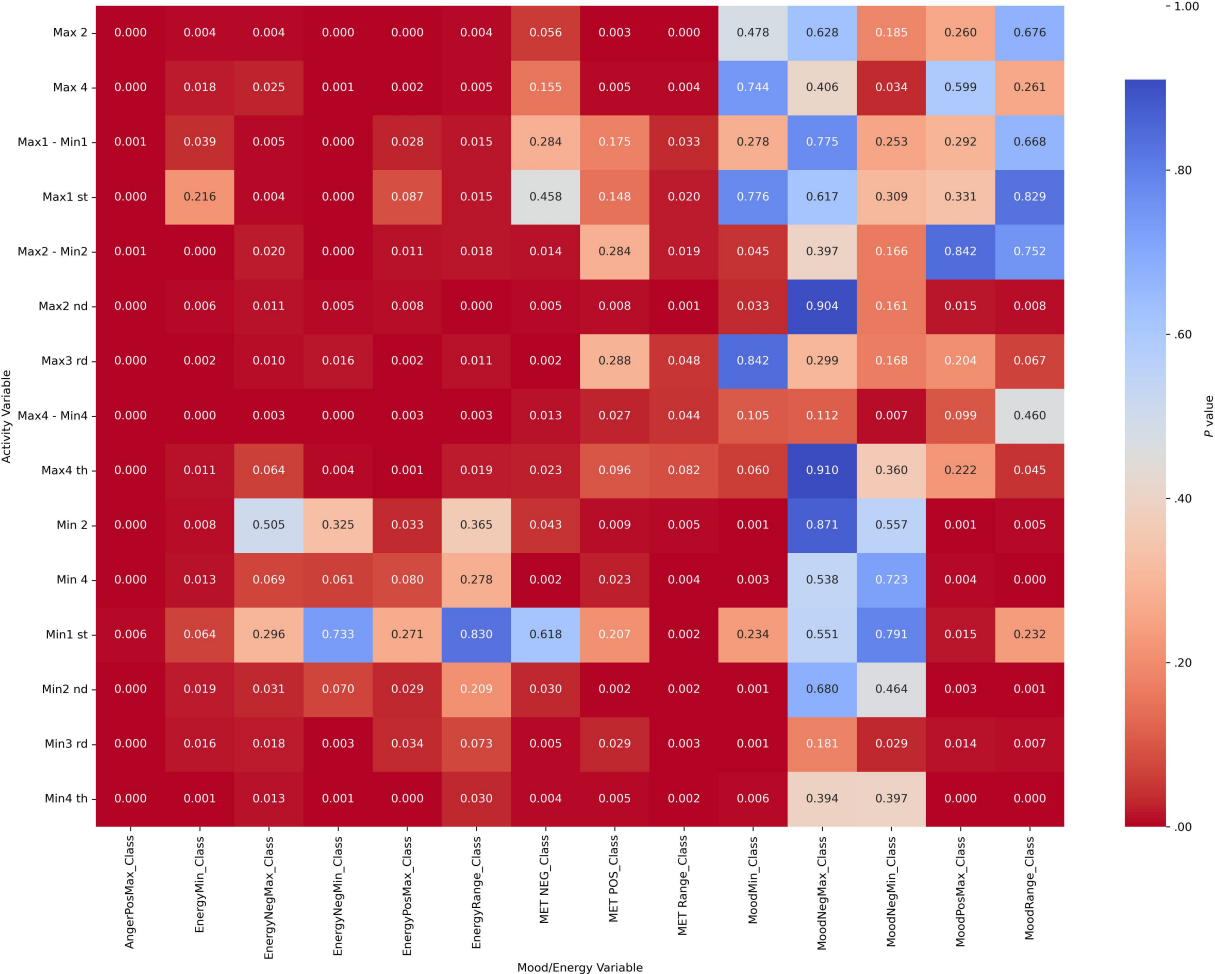
Heatmap visualization to illustrate the *P* values of activity (actigraphy quartiles: Max1-Max4 and Min1-Min4; “Max1…Max4” and “Min1…Min4” refer to quartiles of 4 nonoverlapping 60-min peak/trough windows per day [07:00 AM-09:59 PM]; 1=lowest quartile, 4=highest quartile) and mood/energy category (MoodPosMax/Min, MoodNegMax/Min, EnergyPosMax/Min, EnergyNegMax/Min, and range=daily max-min) correlations for other diagnoses. Max: maximum; MET: Mood and Energy Thermometer; Min: minimum; Neg: negative; Pos: positive.

**Figure 5.**
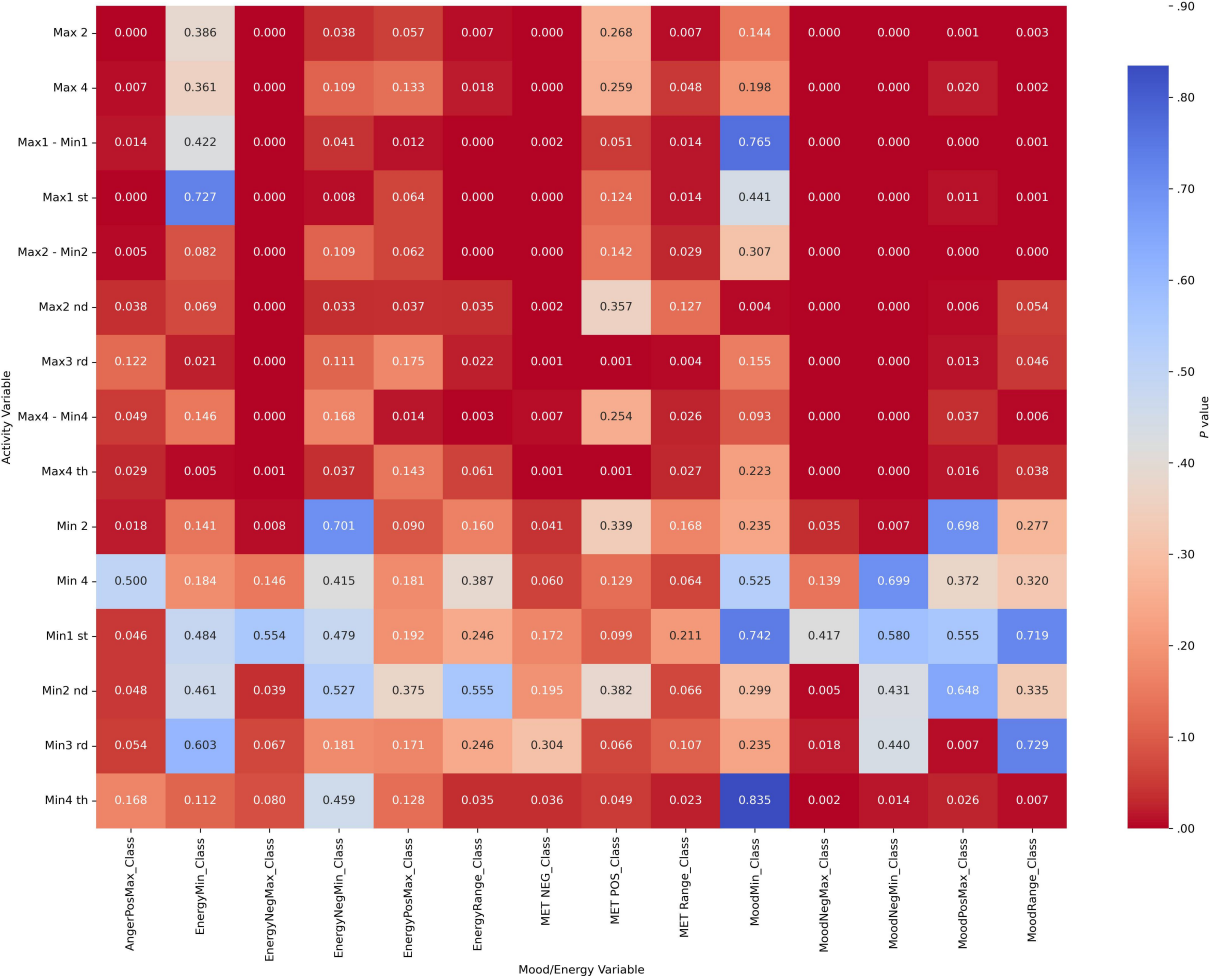
Heatmap visualization to illustrate the *P* values of activity (actigraphy quartiles: Max1-Max4 and Min1-Min4; “Max1…Max4” and “Min1…Min4” refer to quartiles of 4 nonoverlapping 60-min peak/trough windows per day [07:00 AM-09:59 PM]; 1=lowest quartile, 4=highest quartile) and mood/energy category (MoodPosMax/Min, MoodNegMax/Min, EnergyPosMax/Min, EnergyNegMax/Min, and range=daily max-min) correlations for bipolar disorder without attention-deficit/hyperactivity disorder. Max: maximum; MET: Mood and Energy Thermometer; Min: minimum; Neg: negative; Pos: positive.

#### Part C: Effect Size Analyses (Cramér V)

**Figure 6.**
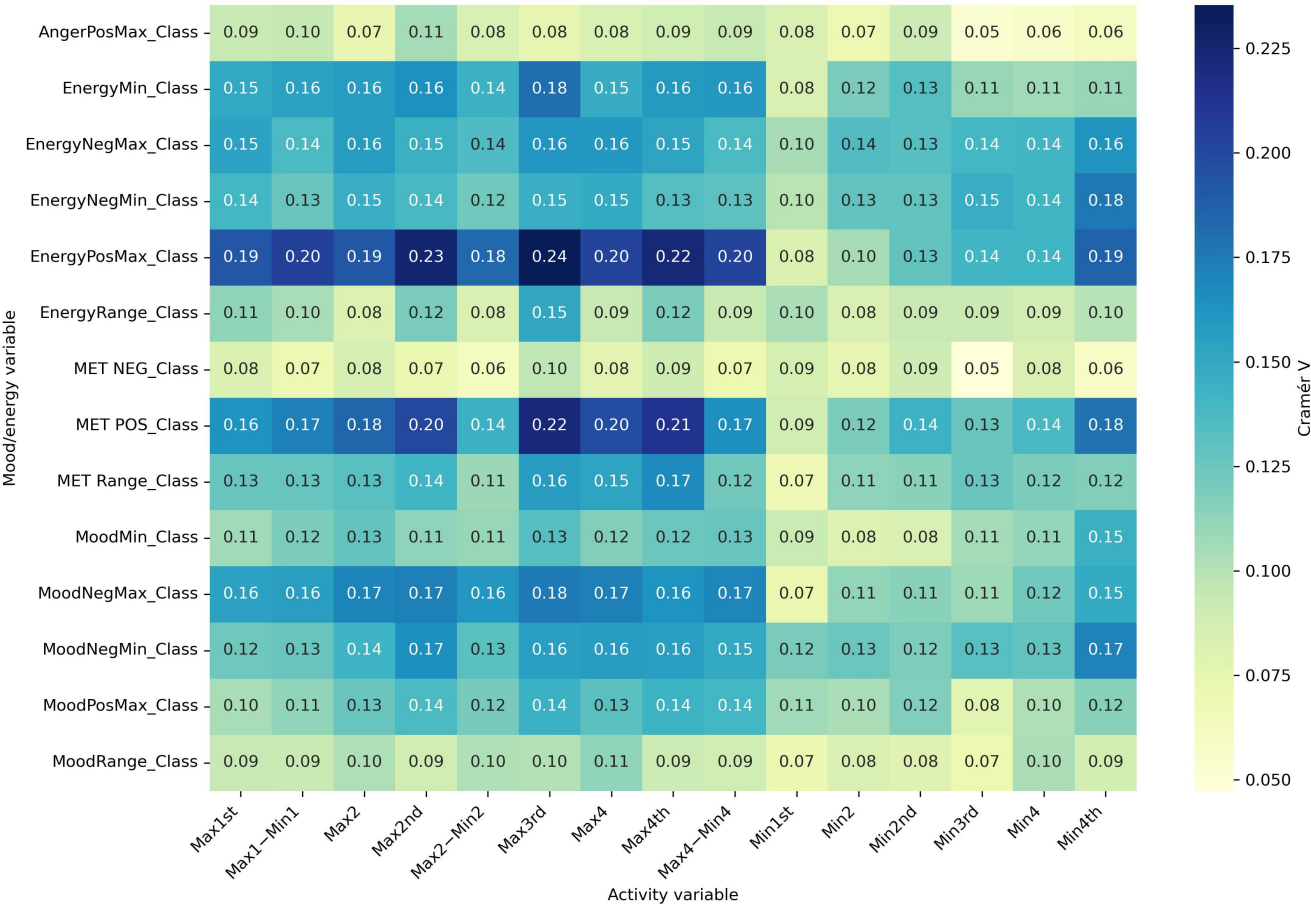
Cramér V heatmap for attention-deficit/hyperactivity disorder without bipolar disorder. Associations between actigraphy quartiles (rows: Max1-Max4 and Min1-Min4; “Max1…Max4” and “Min1…Min4” refer to quartiles of 4 nonoverlapping 60-min peak/trough windows per day [07:00 AM-09:59 PM]; 1=lowest quartile, 4=highest quartile) and Mood and Energy Thermometer (MET) mood/energy severity categories (columns: MoodPosMax/Min, MoodNegMax/Min, EnergyPosMax/Min, EnergyNegMax/Min, and range=daily max-min). The “Class” suffix indicates MET severity bins: OK (<3), mild (3-4), moderate (5-6), and severe (>6). A larger Cramér V value denotes a stronger association (approximately 0.10 is weak; approximately 0.20 is moderate). Max: maximum; Min: minimum; Neg: negative; Pos: positive.

**Figure 7.**
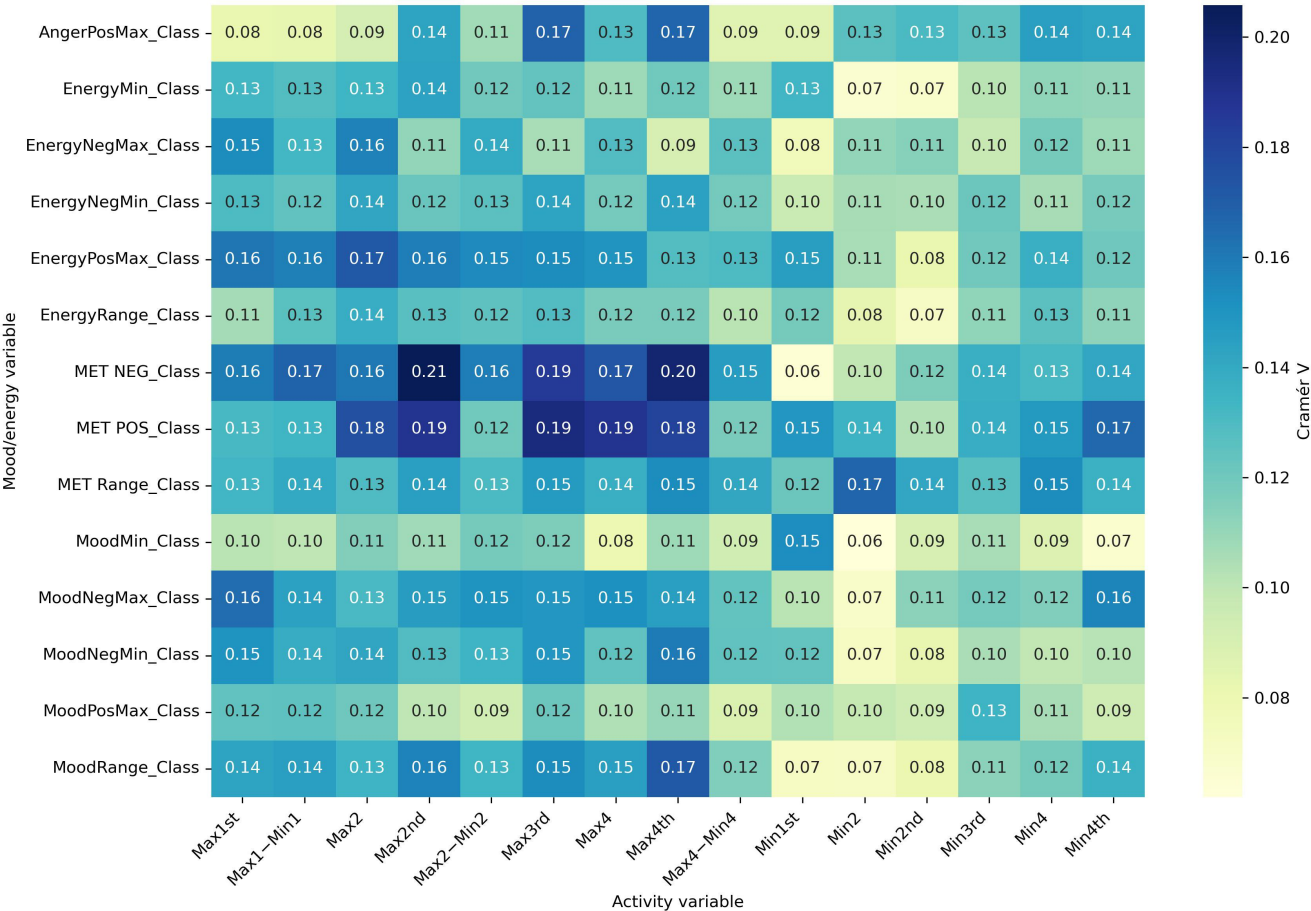
Cramér V heatmap for bipolar disorder with attention-deficit/hyperactivity disorder. Associations between actigraphy quartiles (rows: Max1-Max4 and Min1-Min4; “Max1…Max4” and “Min1…Min4” refer to quartiles of 4 nonoverlapping 60-min peak/trough windows per day [07:00 AM-09:59 PM]; 1=lowest quartile, 4=highest quartile) and Mood and Energy Thermometer (MET) mood/energy severity categories (columns: MoodPosMax/Min, MoodNegMax/Min, EnergyPosMax/Min, EnergyNegMax/Min, and range=daily max-min). The “Class” suffix indicates MET severity bins: OK (<3), mild (3-4), moderate (5-6), and severe (>6). A larger Cramér V value denotes a stronger association (approximately 0.10 is weak; approximately 0.20 is moderate). Max: maximum; Min: minimum; Neg: negative; Pos: positive.

**Figure 8.**
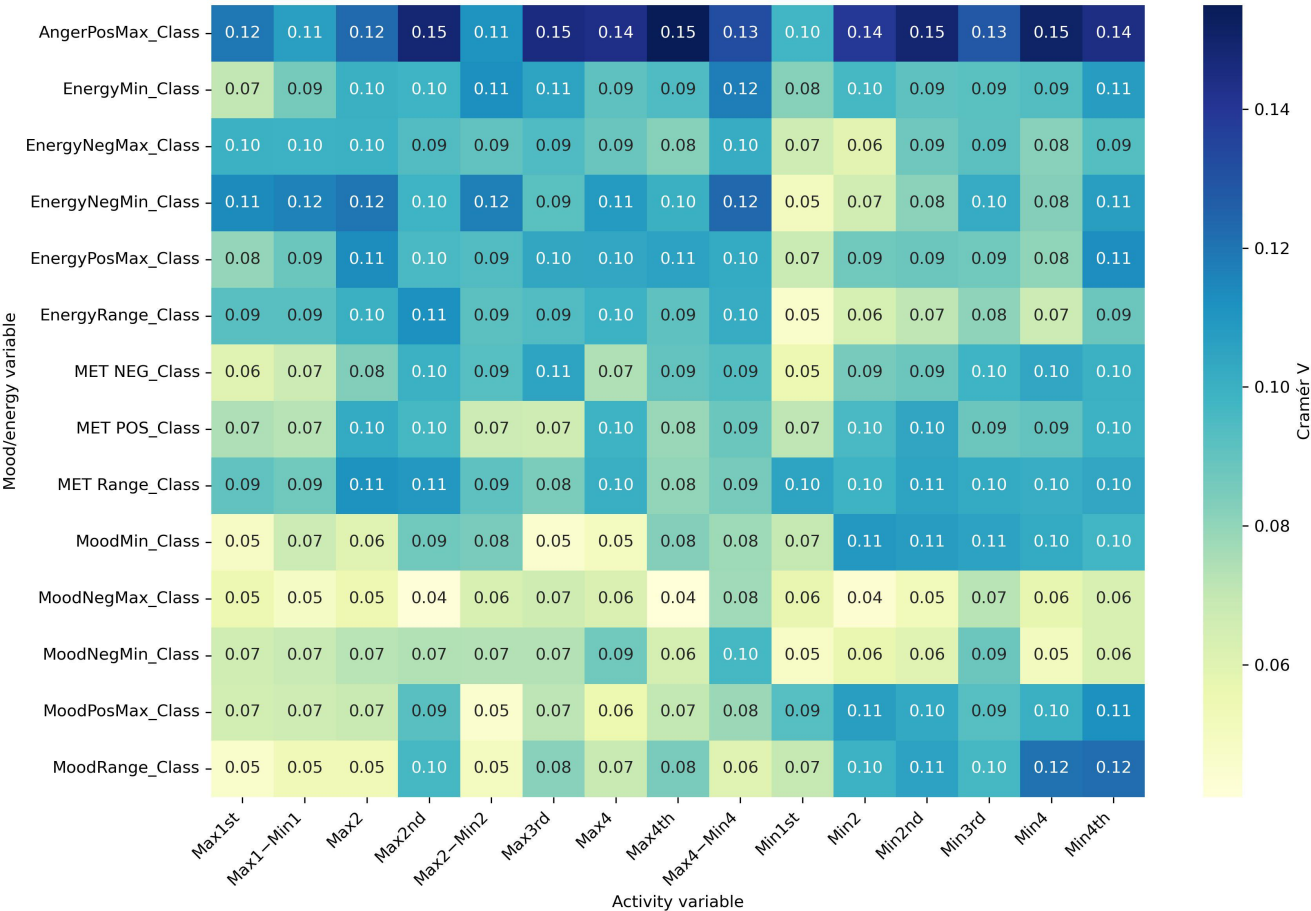
Cramér V heatmap for other diagnoses. Associations between actigraphy quartiles (rows: Max1-Max4 and Min1-Min4; “Max1…Max4” and “Min1…Min4” refer to quartiles of 4 nonoverlapping 60-min peak/trough windows per day [07:00 AM-09:59 PM]; 1=lowest quartile, 4=highest quartile) and Mood and Energy Thermometer (MET) mood/energy severity categories (columns: MoodPosMax/Min, MoodNegMax/Min, EnergyPosMax/Min, EnergyNegMax/Min, and range=daily max-min). The “Class” suffix indicates MET severity bins: OK (<3), mild (3-4), moderate (5-6), and severe (>6). A larger Cramér V value denotes a stronger association (approximately 0.10 is weak; approximately 0.20 is moderate). Max: maximum; Min: minimum; Neg: negative; Pos: positive.

**Figure 9.**
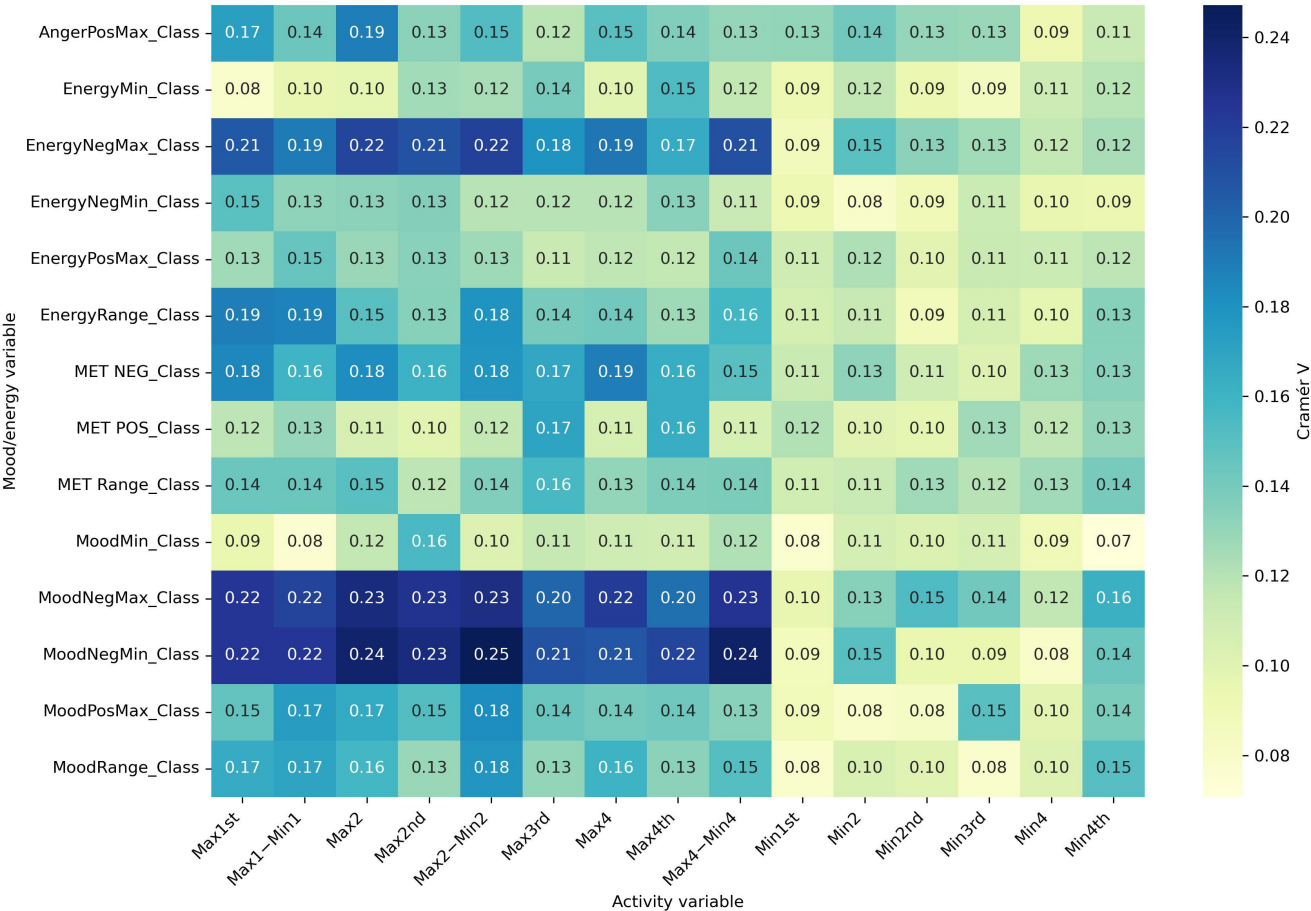
Cramér V heatmap for bipolar disorder without attention-deficit/hyperactivity disorder. Associations between actigraphy quartiles (rows: Max1-Max4 and Min1-Min4; “Max1…Max4” and “Min1…Min4” refer to quartiles of 4 nonoverlapping 60-min peak/trough windows per day [07:00 AM-09:59 PM]; 1=lowest quartile, 4=highest quartile) and Mood and Energy Thermometer (MET) mood/energy severity categories (columns: MoodPosMax/Min, MoodNegMax/Min, EnergyPosMax/Min, EnergyNegMax/Min, and range=daily max-min). The “Class” suffix indicates MET severity bins: OK (<3), mild (3-4), moderate (5-6), and severe (>6). A larger Cramér V value denotes a stronger association (approximately 0.10 is weak; approximately 0.20 is moderate). Max: maximum; Min: minimum; Neg: negative; Pos: positive.

Effect size analyses using Cramér V demonstrated diagnostic specificity in the strength of associations (Figures 6-9). Positive mood states (MoodPosMax_Class) showed weak associations with activity across all groups (V=0.065‐0.173), with the strongest observed in the “BD without ADHD” group (V=0.173). Negative mood states (MoodNegMax_Class) exhibited stronger associations, particularly in the “BD without ADHD” group (V=0.195‐0.241). For energy-related variables, positive energy (EnergyPosMax_Class) demonstrated moderate associations in the “ADHD without BD” group (V=0.225), while negative energy (EnergyNegMax_Class) was moderately associated with activity in the “BD without ADHD” group (V=0.216). Variability measures also differed. Mood variability (MoodRange_Class) showed weak associations overall, whereas energy variability (EnergyRange_Class) exhibited moderate associations in the “BD without ADHD” group (V=0.186).

Together, these analyses suggest that “BD without ADHD” is characterized by tighter coupling between negative affect and extreme activity fluctuations, whereas “ADHD without BD” is more strongly associated with high activity and elevated positive energy.

### AI Modeling of the Prediction of Mood Mean by Using Energy and Actigraphy Features

#### AI Classification of Daily Mood Severity (Leak-Safe Pipeline)

We reframed the AI analysis as binary classification with patient-wise validation to avoid leakage. Two mood-only outcomes were defined from the MET: SevereDay (same day) and SevereTomorrow (next day), labeled severe if MoodPosMax ≥7 or MoodNegMax ≥7. Energy variables were used only as predictors (not in labels).

#### Data and Class Balance

The data and class balance for SevereDay were as follows: 0, 1368; 1, 613 (n=1981 days), and that for SevereTomorrow was as follows: 0, 1218; 1, 554 (n=1772 day-pairs).

#### Models and Validation

We compared logistic regression, HistGradientBoosting, random forest, extra trees, and XGBoost (when available) plus a soft-vote ensemble. Preprocessing (median imputation, one-hot encoding, and scaling, when needed) and the SMOTE were performed inside the pipeline on training folds only. Generalization was estimated with 5-fold GroupKFold (patient as group). Inner RandomizedSearchCV (3-fold GroupKFold) tuned hyperparameters using ROC-AUC. A held-out, group-aware validation split within each training fold optimized the decision threshold (sweep 0.05‐0.95) for accuracy (we also report *F*_1_-score).

#### Primary Metrics (Mean Across Folds and Tuned Thresholds)

Logistic regression was the best model for SevereDay (same day; ROC-AUC=0.842; PR-AUC=0.704; accuracy=0.777; *F*_1_-score=0.656) and SevereTomorrow (next day; ROC-AUC=0.780; PR-AUC=0.622; accuracy=0.748; *F*_1_-score=0.562) (Figure 1).

#### Final-Fold Snapshots (Best Model)

The findings for SevereDay were as follows: ROC-AUC=0.849; PR-AUC=0.702; accuracy=0.791; *F*_1_-score=0.673 (severe class: precision=0.61; recall=0.75), and those for SevereTomorrow were as follows: ROC-AUC=0.796; PR-AUC=0.612; accuracy=0.788; *F*_1_-score=0.599 (severe class: precision=0.67; recall=0.54).

**Figure 10.**
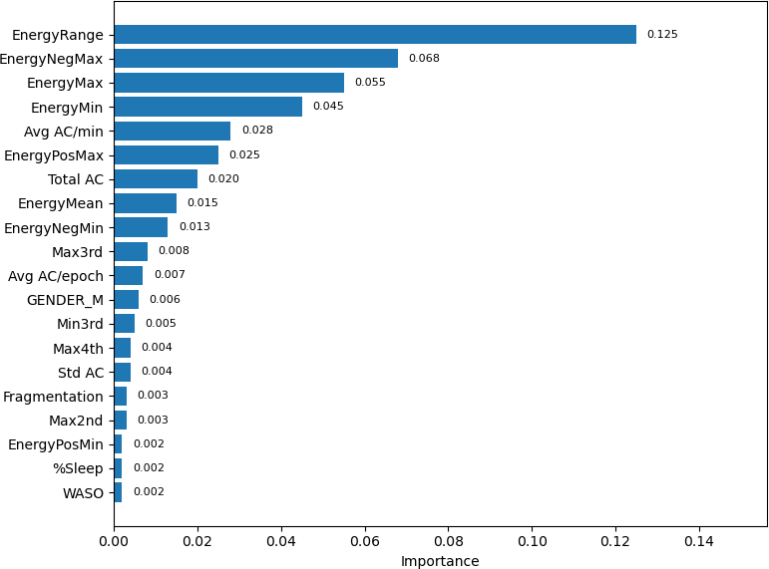
Feature importance for predicting next-day severe mood (SevereTomorrow). Top 20 permutation feature importances for the SevereTomorrow classifier (logistic regression), averaged across 5-fold patient-wise cross-validation. Bars show the mean decrease in performance when permuting each feature, indicating its relative predictive contribution. Key predictors were energy-derived features (EnergyRange, EnergyNegMax, EnergyMax, EnergyMin, and EnergyMean), followed by actigraphy features (average AC/min, average AC/epoch, total AC, standard AC, and maximum AC) and selected sleep metrics (efficiency, percentage sleep, wake after sleep onset [WASO], and fragmentation). The prefix “num” refers to “numeric predictor,” and the prefix “cat” refers to “one-hot categorical predictor.” “Max1…Max4” and “Min1…Min4” refer to quartiles of 4 nonoverlapping 60-min peak/trough windows per day (1=lowest quartile, 4=highest quartile). AC: activity count; Avg: average; Max: maximum; Min: minimum; Neg: negative; Pos: positive.

Cross-validated permutation importance (Figure 10) revealed that energy-derived features were the strongest predictors, particularly EnergyRange, EnergyNegMax, EnergyMax, EnergyMin, and EnergyMean. Additional contributions came from activity features (average AC/min, average AC/epoch, total AC, and extremes) and select sleep variables such as efficiency.

## Discussion

### Overview of Key Findings

This study introduces a novel methodological approach by transforming continuous actigraphy data into quartiles and applying categorical statistical analyses to examine associations among physical activity, mood, and energy in adolescents with BD, ADHD, and other psychiatric diagnoses. Our results revealed significant diagnostic differences in self-reported mood and energy profiles, with distinct patterns of variability and correlation strength across groups. In particular, BD without ADHD was characterized by negative mood-energy fluctuations tied to higher ACs, while ADHD without BD showed a stronger link between activity levels and positive energy states.

### Mood-Energy Coupling Across Diagnostic Groups

The correlation analyses demonstrated strong within-domain associations—positive mood with positive energy and negative mood with negative energy—consistent with prior research showing that mood and energy are closely interdependent in pediatric populations [15]. The ADHD without BD group exhibited a particularly high correlation (*r*=0.78) between maximum positive mood and maximum positive energy, underscoring the pronounced interplay between these dimensions in the absence of comorbid BD [16,17]. In contrast, BD without ADHD displayed the strongest associations between negative mood, negative energy, and activity fluctuations (Cramér V=0.195‐0.241). The presence of comorbid ADHD in BD significantly impacts mood and energy dynamics, suggesting distinct clinical phenotypes resulting from this co-occurrence [17]. This aligns with prior research showing that mood instability in BD is often accompanied by circadian disruptions and irregular activity levels [5,7,8]. These findings suggest that actigraphy may serve as an objective marker of negative affect dysregulation in BD. Moreover, the moderate association between energy variability and actigraphy indicates that daily energy fluctuations may provide a more reliable marker of dysregulation than self-reported mood alone.

In ADHD without BD, higher activity levels were more strongly associated with positive energy (Cramér V=0.225), suggesting that actigraphy reflects arousal and hyperactivity rather than mood instability. This aligns with prior work differentiating ADHD-driven hyperactivity from BD-related mood changes [17]. Features, such as EnergyMean, EnergyMax/Min, and average ACs, emerged as potential diagnostic markers to distinguish ADHD from BD in clinical settings.

Patients classified under “other diagnoses” showed the greatest variability in negative mood and energy, highlighting instability but weaker overall associations with actigraphy. This may reflect the heterogeneity of this group, which included depressive and disruptive disorders, where actigraphy may be less informative for mood-energy dynamics [18-20]. This aligns with findings that indicate a spectrum of mood dysregulation that extends beyond traditional diagnostic boundaries, necessitating a more comprehensive understanding of the interplay between physical activity and psychological factors [21]. Our findings emphasize the complex interplay among physical activity, mood, and energy levels in pediatric psychiatric conditions [22]. These findings are consistent with previous research [12,23], emphasizing the utility of actigraphy in assessing sleep disorders, evaluating circadian rhythms, and pediatric sleep research. Incorporating mood, energy, and actigraphy assessments can refine clinical phenotyping and enable tailored interventions targeting specific symptom dimensions in each disorder [24-26].

### Machine Learning Prediction of Severe Mood States

Machine learning results demonstrated that combining actigraphy, sleep, and energy features can meaningfully classify mood severity. Our leak-safe pipeline showed that same-day severe mood could be predicted with good accuracy (ROC-AUC=0.84), while next-day risk was more modest but clinically useful (ROC-AUC=0.78). Energy variability, peak values, and average actigraphy metrics consistently ranked among the most important predictors. Notably, a regularized linear model (logistic regression) performed on par with or better than tree-based ensembles, suggesting that much of the predictive signal is captured by summary features when leakage is controlled. At clinically relevant thresholds, the same-day model achieved severe-class recall of approximately 0.75 with precision of approximately 0.61, supporting its potential for inpatient monitoring. Next-day detection (recall of approximately 0.54; precision of approximately 0.67) may provide actionable early alerts for staffing or clinical check-ins.

### Clinical Implications

Taken together, these findings suggest that wearable-derived actigraphy and sleep measures, when combined with simple daily energy ratings, provide a robust framework for real-time monitoring of mood severity in adolescents with psychiatric conditions [27]. BD without ADHD emerges as a group where actigraphy is particularly informative [28] for identifying negative mood states, while ADHD without BD is better characterized by associations with hyperactivity and positive energy. Clinically, these results support incorporating daily energy tracking into psychiatric assessments, as energy may be a more stable and discriminative marker than mood in certain contexts.

### Limitations and Future Directions

This study has several limitations. First, its cross-sectional design precludes causal inferences. Longitudinal studies are needed to clarify whether activity fluctuations drive mood changes or vice versa. Second, actigraphy alone does not capture contextual influences, such as medication effects, sleep disturbances, and environmental stressors. While our naturalistic inpatient setting increases ecological validity, future research should integrate multimodal assessments, including polysomnography, ecological momentary assessment, and biomarker data to better account for these factors. Third, class imbalance in severe mood states may have reduced statistical power. Although our group-aware, leak-safe modeling approach mitigated this issue and applied the SMOTE only within training folds, rare-event calibration remains a challenge. Moreover, the models did not incorporate within-patient correlation via mixed-effects or adjust for treatment and milieu variables, which are both priorities for future work.

### Comparison With the SMOTE-Based Model

A prior SMOTE-based approach balanced multiclass mood and energy categories (OK, mild, moderate, and severe) but did not enforce patient-wise separation, raising concerns about data leakage. Our revised framework addressed this by restricting analyses to binary severity outcomes, applying the SMOTE only within training folds, and using group-level cross-validation. This design yields more conservative, leakage-safe estimates of real-world performance.

### Conclusion

Our findings highlight disorder-specific patterns of mood-energy dysregulation: BD without ADHD was characterized by strong associations between negative mood or energy states and extreme activity fluctuations, while ADHD without BD was characterized by high activity linked to positive energy rather than mood instability. In a leak-safe, patient-wise framework, combining daily energy ratings with actigraphy- and sleep-derived features reliably identified same-day severe mood and provided moderate next-day risk signals (ROC-AUC=0.84 and 0.78, respectively). Energy variability and peaks, alongside average and extreme activity metrics, consistently emerged as the most informative predictors.

Clinically, these results support incorporating daily energy tracking with wearable devices into psychiatric assessments. Actigraphy may be especially valuable for identifying negative affect states in BD, while energy ratings offer a stable and discriminative marker across conditions. The integration of objective activity measures with subjective energy assessments could refine real-time psychiatric monitoring, improve diagnostic precision, and guide personalized interventions for adolescents with BD, ADHD, and related disorders.

## Supplementary material

10.2196/78163 Multimedia Appendix 1 Box plot comparisons providing a visual representation of the distribution of mood and energy variables across the diagnostic labels (bipolar disorder [BD] without attention-deficit/hyperactivity disorder [ADHD]: blue; ADHD without BD: yellow; BD with ADHD: green; other diagnoses: red). Significant pairwise comparisons are indicated by an arrow.

10.2196/78163 Multimedia Appendix 2 Pairwise Mann-Whitney *U* comparisons of mood and energy variables across diagnostic groups.

10.2196/78163 Multimedia Appendix 3 Density plots of mood and energy variables across diagnostic groups.

10.2196/78163 Multimedia Appendix 4 Variability metrics for mood and energy variables across diagnostic groups.

10.2196/78163 Multimedia Appendix 5 Correlation matrix between actigraphy-based activity quartiles and self-reported mood and energy variables.

## Abbreviations

AC: activity count
ADHD: attention-deficit/hyperactivity disorder
AI: artificial intelligence
BD: bipolar disorder
DSM-5: Diagnostic and Statistical Manual of Mental Disorders, Fifth Edition
HistGradientBoosting: histogram-based gradient boosting
MET: Mood and Energy Thermometer
PR-AUC: precision-recall area under the curve
ROC-AUC: receiver operating characteristic area under the curve
SMOTE: Synthetic Minority Oversampling Technique
XGBoost: extreme gradient boosting
