# Supplementary figures and images for: Differentiating Pediatric Bipolar Disorder, Attention-Deficit/Hyperactivity Disorder, and Other Psychopathologies Using Self-Reported Mood and Energy Data and Actigraphy Findings: Correlation and Machine Learning–Based Prediction of Mood Severity
Source: JMIR Ment Health. 2025 Dec 4;12:e78163. doi: 10.2196/78163 (PMC12677876; doi:10.2196/78163)

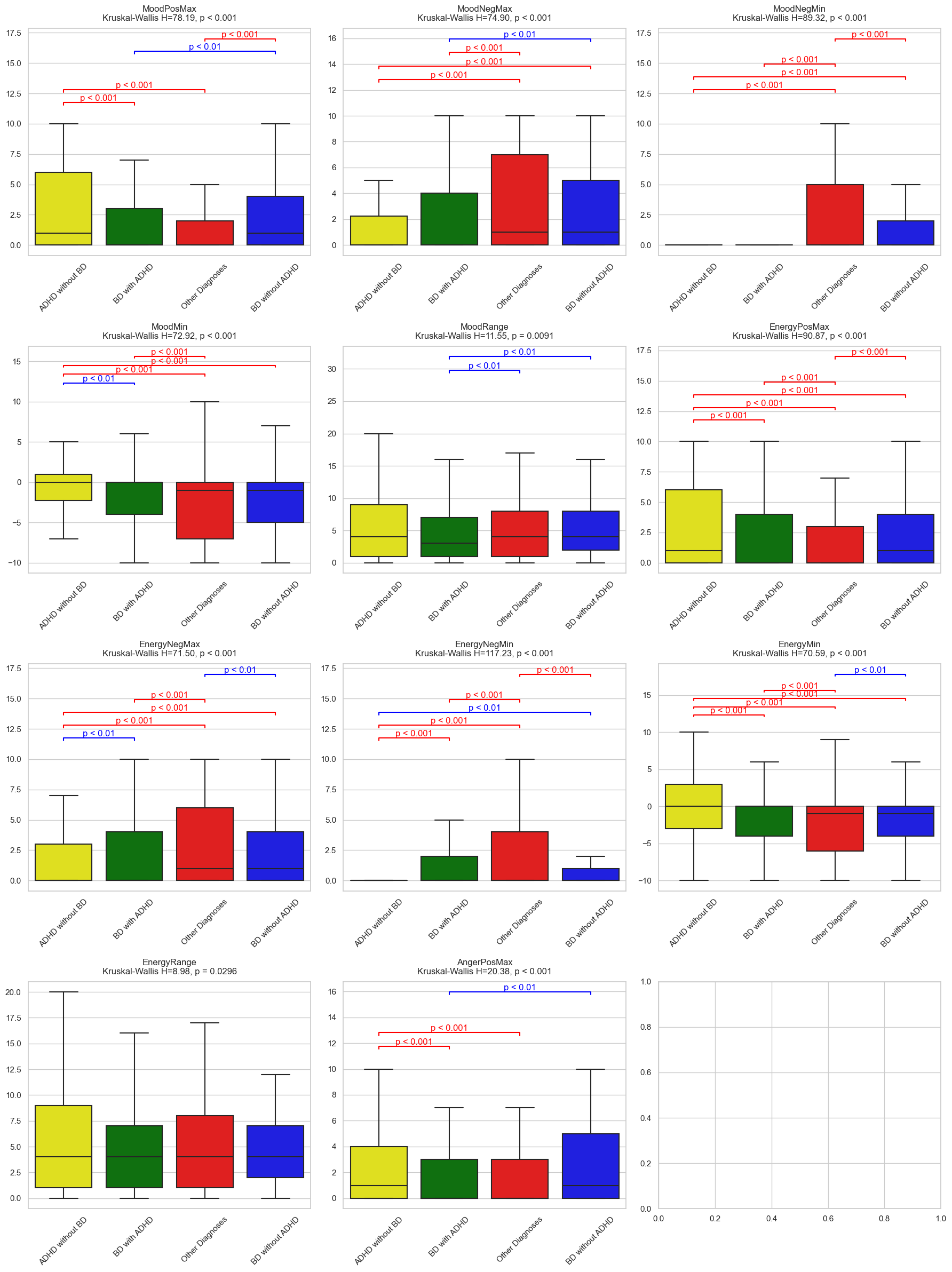

Supplement: Multimedia Appendix 1 [file mental-v12-e78163-s001.docx]
