# Supplementary material for: Differentiating Pediatric Bipolar Disorder, Attention-Deficit/Hyperactivity Disorder, and Other Psychopathologies Using Self-Reported Mood and Energy Data and Actigraphy Findings: Correlation and Machine Learning–Based Prediction of Mood Severity
Source: JMIR Ment Health. 2025 Dec 4;12:e78163. doi: 10.2196/78163 (PMC12677876; doi:10.2196/78163)
Supplement: Multimedia Appendix 2 [file mental-v12-e78163-s002.docx]

**Multimedia Appendix 2.** Pairwise Mann-Whitney *U* comparisons of mood and energy variables across diagnostic groups.

Results of pairwise Mann-Whitney U tests comparing mood and energy self-report variables between diagnostic groups (BD without ADHD, BD with ADHD, ADHD without BD, and Other Diagnoses). P-values were adjusted for multiple comparisons using Bonferroni correction (p_corrected < 0.004). Significant differences are highlighted, with the directionality (higher group) specified. These results confirm distinct mood and energy severity profiles across diagnostic categories, supporting the need for stratified modeling approaches such as SMOTE.

| **Variable** | **Group 1** | **Group 2** | **p-value** | **Significant** | **Higher Group** | **Significance Label** |
| --- | --- | --- | --- | --- | --- | --- |
| MoodPosMax | ADHD without BD | BD with ADHD | 3.28148E-06 | TRUE | ADHD without BD | p < 0.001 |
| MoodPosMax | ADHD without BD | Other Diagnoses | 1.17522E-16 | TRUE | ADHD without BD | p < 0.001 |
| MoodPosMax | ADHD without BD | BD without ADHD | 0.107674368 | FALSE | ADHD without BD | p > 0.05 |
| MoodPosMax | BD with ADHD | Other Diagnoses | 0.008582333 | FALSE | BD with ADHD | p < 0.01 |
| MoodPosMax | BD with ADHD | BD without ADHD | 0.005468682 | TRUE | BD without ADHD | p < 0.01 |
| MoodPosMax | Other Diagnoses | BD without ADHD | 1.03732E-08 | TRUE | BD without ADHD | p < 0.001 |
| MoodNegMax | ADHD without BD | BD with ADHD | 0.018713454 | FALSE | BD with ADHD | p < 0.05 |
| MoodNegMax | ADHD without BD | Other Diagnoses | 1.60461E-15 | TRUE | Other Diagnoses | p < 0.001 |
| MoodNegMax | ADHD without BD | BD without ADHD | 1.94E-07 | TRUE | BD without ADHD | p < 0.001 |
| MoodNegMax | BD with ADHD | Other Diagnoses | 1.71996E-07 | TRUE | Other Diagnoses | p < 0.001 |
| MoodNegMax | BD with ADHD | BD without ADHD | 4.55E-03 | TRUE | BD without ADHD | p < 0.01 |
| MoodNegMax | Other Diagnoses | BD without ADHD | 4.65E-02 | FALSE | Other Diagnoses | p < 0.05 |
| MoodNegMin | ADHD without BD | BD with ADHD | 0.094979223 | FALSE | BD with ADHD | p > 0.05 |
| MoodNegMin | ADHD without BD | Other Diagnoses | 2.64914E-17 | TRUE | Other Diagnoses | p < 0.001 |
| MoodNegMin | ADHD without BD | BD without ADHD | 1.08059E-05 | TRUE | BD without ADHD | p < 0.001 |
| MoodNegMin | BD with ADHD | Other Diagnoses | 3.03635E-10 | TRUE | Other Diagnoses | p < 0.001 |
| MoodNegMin | BD with ADHD | BD without ADHD | 0.013152686 | FALSE | BD without ADHD | p < 0.05 |
| MoodNegMin | Other Diagnoses | BD without ADHD | 0.000563975 | TRUE | Other Diagnoses | p < 0.001 |
| MoodMin | ADHD without BD | BD with ADHD | 4.58E-03 | TRUE | ADHD without BD | p < 0.01 |
| MoodMin | ADHD without BD | Other Diagnoses | 8.89135E-16 | TRUE | ADHD without BD | p < 0.001 |
| MoodMin | ADHD without BD | BD without ADHD | 1.83E-06 | TRUE | ADHD without BD | p < 0.001 |
| MoodMin | BD with ADHD | Other Diagnoses | 9.13075E-07 | TRUE | BD with ADHD | p < 0.001 |
| MoodMin | BD with ADHD | BD without ADHD | 2.74E-02 | FALSE | BD with ADHD | p < 0.05 |
| MoodMin | Other Diagnoses | BD without ADHD | 2.38E-02 | FALSE | BD without ADHD | p < 0.05 |
| MoodRange | ADHD without BD | BD with ADHD | 0.042299931 | FALSE | ADHD without BD | p < 0.05 |
| MoodRange | ADHD without BD | Other Diagnoses | 0.558295647 | FALSE | ADHD without BD | p > 0.05 |
| MoodRange | ADHD without BD | BD without ADHD | 0.268026999 | FALSE | BD without ADHD | p > 0.05 |
| MoodRange | BD with ADHD | Other Diagnoses | 0.002511799 | TRUE | Other Diagnoses | p < 0.01 |
| MoodRange | BD with ADHD | BD without ADHD | 2.15E-03 | TRUE | BD without ADHD | p < 0.01 |
| MoodRange | Other Diagnoses | BD without ADHD | 5.44E-01 | FALSE | BD without ADHD | p > 0.05 |
| EnergyPosMax | ADHD without BD | BD with ADHD | 1.31922E-06 | TRUE | ADHD without BD | p < 0.001 |
| EnergyPosMax | ADHD without BD | Other Diagnoses | 6.51E-21 | TRUE | ADHD without BD | p < 0.001 |
| EnergyPosMax | ADHD without BD | BD without ADHD | 0.000290502 | TRUE | ADHD without BD | p < 0.001 |
| EnergyPosMax | BD with ADHD | Other Diagnoses | 0.000427398 | TRUE | BD with ADHD | p < 0.001 |
| EnergyPosMax | BD with ADHD | BD without ADHD | 3.40E-01 | FALSE | BD with ADHD | p > 0.05 |
| EnergyPosMax | Other Diagnoses | BD without ADHD | 3.30E-06 | TRUE | BD without ADHD | p < 0.001 |
| EnergyNegMax | ADHD without BD | BD with ADHD | 0.005965137 | TRUE | BD with ADHD | p < 0.01 |
| EnergyNegMax | ADHD without BD | Other Diagnoses | 4.92887E-16 | TRUE | Other Diagnoses | p < 0.001 |
| EnergyNegMax | ADHD without BD | BD without ADHD | 2.80924E-05 | TRUE | BD without ADHD | p < 0.001 |
| EnergyNegMax | BD with ADHD | Other Diagnoses | 1.83E-06 | TRUE | Other Diagnoses | p < 0.001 |
| EnergyNegMax | BD with ADHD | BD without ADHD | 2.34E-01 | FALSE | BD without ADHD | p > 0.05 |
| EnergyNegMax | Other Diagnoses | BD without ADHD | 0.002017389 | TRUE | Other Diagnoses | p < 0.01 |
| EnergyNegMin | ADHD without BD | BD with ADHD | 2.67E-04 | TRUE | BD with ADHD | p < 0.001 |
| EnergyNegMin | ADHD without BD | Other Diagnoses | 1.27616E-23 | TRUE | Other Diagnoses | p < 0.001 |
| EnergyNegMin | ADHD without BD | BD without ADHD | 1.74E-03 | TRUE | BD without ADHD | p < 0.01 |
| EnergyNegMin | BD with ADHD | Other Diagnoses | 4.37336E-09 | TRUE | Other Diagnoses | p < 0.001 |
| EnergyNegMin | BD with ADHD | BD without ADHD | 6.21E-01 | FALSE | BD with ADHD | p > 0.05 |
| EnergyNegMin | Other Diagnoses | BD without ADHD | 5.95E-09 | TRUE | Other Diagnoses | p < 0.001 |
| EnergyMin | ADHD without BD | BD with ADHD | 0.000495136 | TRUE | ADHD without BD | p < 0.001 |
| EnergyMin | ADHD without BD | Other Diagnoses | 5.7996E-16 | TRUE | ADHD without BD | p < 0.001 |
| EnergyMin | ADHD without BD | BD without ADHD | 1.15428E-05 | TRUE | ADHD without BD | p < 0.001 |
| EnergyMin | BD with ADHD | Other Diagnoses | 2.42726E-05 | TRUE | BD with ADHD | p < 0.001 |
| EnergyMin | BD with ADHD | BD without ADHD | 0.337277876 | FALSE | BD with ADHD | p > 0.05 |
| EnergyMin | Other Diagnoses | BD without ADHD | 0.003992989 | TRUE | BD without ADHD | p < 0.01 |
| EnergyRange | ADHD without BD | BD with ADHD | 1.28E-02 | FALSE | ADHD without BD | p < 0.05 |
| EnergyRange | ADHD without BD | Other Diagnoses | 0.338837708 | FALSE | ADHD without BD | p > 0.05 |
| EnergyRange | ADHD without BD | BD without ADHD | 3.42E-02 | FALSE | ADHD without BD | p < 0.05 |
| EnergyRange | BD with ADHD | Other Diagnoses | 0.049644643 | FALSE | Other Diagnoses | p < 0.05 |
| EnergyRange | BD with ADHD | BD without ADHD | 7.85E-01 | FALSE | BD with ADHD | p > 0.05 |
| EnergyRange | Other Diagnoses | BD without ADHD | 1.13E-01 | FALSE | Other Diagnoses | p > 0.05 |
| AngerPosMax | ADHD without BD | BD with ADHD | 2.40E-04 | TRUE | ADHD without BD | p < 0.001 |
| AngerPosMax | ADHD without BD | Other Diagnoses | 0.000927227 | TRUE | ADHD without BD | p < 0.001 |
| AngerPosMax | ADHD without BD | BD without ADHD | 0.896716787 | FALSE | ADHD without BD | p > 0.05 |
| AngerPosMax | BD with ADHD | Other Diagnoses | 0.284647892 | FALSE | Other Diagnoses | p > 0.05 |
| AngerPosMax | BD with ADHD | BD without ADHD | 3.61E-03 | TRUE | BD without ADHD | p < 0.01 |
| AngerPosMax | Other Diagnoses | BD without ADHD | 1.27E-02 | FALSE | BD without ADHD | p < 0.05 |

sTable 1- Mann-Whitney U Test for Pairwise Comparisons and Bonferroni Correction of Target Variables Between the Four Diagnostic Labels.
