# Supplementary material for: Differentiating Pediatric Bipolar Disorder, Attention-Deficit/Hyperactivity Disorder, and Other Psychopathologies Using Self-Reported Mood and Energy Data and Actigraphy Findings: Correlation and Machine Learning–Based Prediction of Mood Severity
Source: JMIR Ment Health. 2025 Dec 4;12:e78163. doi: 10.2196/78163 (PMC12677876; doi:10.2196/78163)
Supplement: Multimedia Appendix 3 [file mental-v12-e78163-s003.docx]

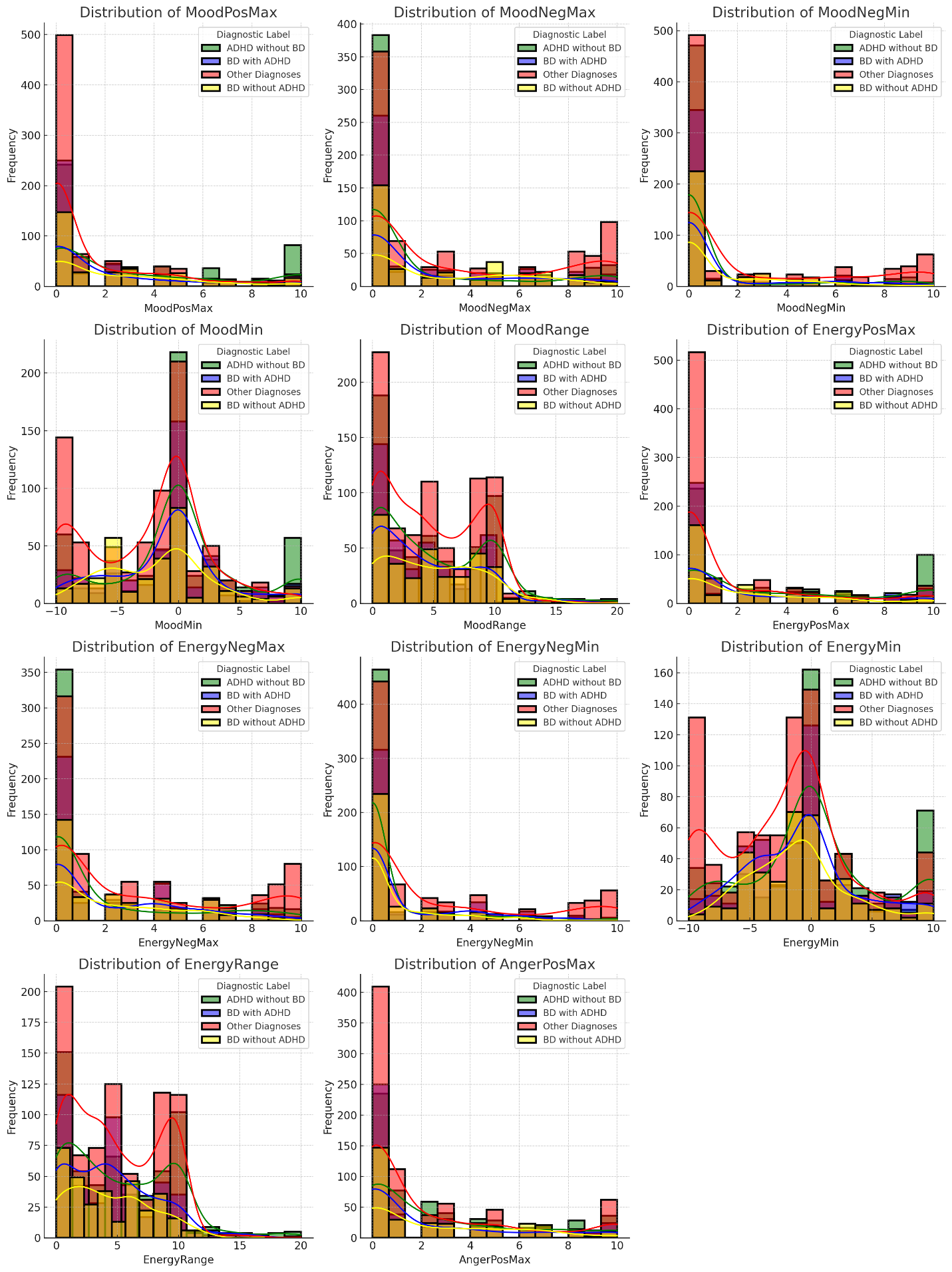


Multimedia Appendix 3. **Density plots of mood and energy variables across diagnostic groups**

Density plots show the distribution of mood- and energy-related variables (MoodPosMax, MoodNegMax, MoodNegMin, MoodMin, MoodRange, EnergyPosMax, EnergyNegMax, EnergyNegMin, EnergyMin, EnergyRange, and AngerPosMax) across diagnostic groups. Most participants clustered in the “OK” range (<3), indicating mild symptoms, while the *ADHD without BD* group showed higher peaks in positive mood and energy, and the *Other Diagnoses* group showed greater density in severe negative mood and energy states.
**Abbreviations:** BD, bipolar disorder.
