# Supplementary material for: Differentiating Pediatric Bipolar Disorder, Attention-Deficit/Hyperactivity Disorder, and Other Psychopathologies Using Self-Reported Mood and Energy Data and Actigraphy Findings: Correlation and Machine Learning–Based Prediction of Mood Severity
Source: JMIR Ment Health. 2025 Dec 4;12:e78163. doi: 10.2196/78163 (PMC12677876; doi:10.2196/78163)
Supplement: Multimedia Appendix 4 [file mental-v12-e78163-s004.docx]

**Multimedia Appendix 4.** Variability metrics for mood and energy variables across diagnostic groups.

|  | **Mean** | **SD** | **Range** | **IQR** | **Variable** |
| --- | --- | --- | --- | --- | --- |
| **ADHD without BD** | 2.980769231 | 3.628740226 | 10 | 6 | MoodPosMax |
| **BD with ADHD** | 2.029279279 | 3.06093841 | 10 | 3 | MoodPosMax |
| **Other Diagnoses** | 1.514392991 | 2.582373372 | 10 | 2 | MoodPosMax |
| **BD without ADHD** | 2.339339339 | 2.862646142 | 10 | 4 | MoodPosMax |
|  |  |  |  |  |  |
| **ADHD without BD** | 1.851398601 | 3.254625936 | 10 | 2.25 | MoodNegMax |
| **BD with ADHD** | 2.155405405 | 3.176620657 | 10 | 4 | MoodNegMax |
| **Other Diagnoses** | 3.260325407 | 3.860969192 | 10 | 7 | MoodNegMax |
| **BD without ADHD** | 2.624624625 | 3.070496054 | 10 | 5 | MoodNegMax |
|  |  |  |  |  |  |
| **ADHD without BD** | 1.008741259 | 2.526551228 | 10 | 0 | MoodNegMin |
| **BD with ADHD** | 1.173423423 | 2.534478453 | 10 | 0 | MoodNegMin |
| **Other Diagnoses** | 2.40175219 | 3.589185502 | 10 | 5 | MoodNegMin |
| **BD without ADHD** | 1.3003003 | 2.270800752 | 10 | 2 | MoodNegMin |
|  |  |  |  |  |  |
| **ADHD without BD** | -0.25 | 5.131075272 | 20 | 3.25 | MoodMin |
| **BD with ADHD** | -1.11036036 | 4.533196427 | 20 | 4 | MoodMin |
| **Other Diagnoses** | -2.524405507 | 4.844353623 | 20 | 7 | MoodMin |
| **BD without ADHD** | -1.6996997 | 4.398099935 | 20 | 5 | MoodMin |
|  |  |  |  |  |  |
| **ADHD without BD** | 4.832167832 | 4.471722422 | 20 | 8 | MoodRange |
| **BD with ADHD** | 4.184684685 | 3.943980142 | 19 | 6 | MoodRange |
| **Other Diagnoses** | 4.774718398 | 3.870628638 | 20 | 7 | MoodRange |
| **BD without ADHD** | 4.963963964 | 3.899168728 | 20 | 6 | MoodRange |
|  |  |  |  |  |  |
| **ADHD without BD** | 3.398601399 | 3.864862341 | 10 | 6 | EnergyPosMax |
| **BD with ADHD** | 2.34009009 | 3.303205365 | 10 | 4 | EnergyPosMax |
| **Other Diagnoses** | 1.675844806 | 2.908600117 | 10 | 3 | EnergyPosMax |
| **BD without ADHD** | 2.27027027 | 2.920469757 | 10 | 4 | EnergyPosMax |
|  |  |  |  |  |  |
| **ADHD without BD** | 1.84965035 | 3.018062939 | 10 | 3 | EnergyNegMax |
| **BD with ADHD** | 2.150900901 | 2.779678611 | 10 | 4 | EnergyNegMax |
| **Other Diagnoses** | 3.182728411 | 3.647590211 | 10 | 6 | EnergyNegMax |
| **BD without ADHD** | 2.162162162 | 2.500146528 | 10 | 4 | EnergyNegMax |
|  |  |  |  |  |  |
| **ADHD without BD** | 0.818181818 | 2.027434823 | 10 | 0 | EnergyNegMin |
| **BD with ADHD** | 1.177927928 | 2.164219446 | 10 | 2 | EnergyNegMin |
| **Other Diagnoses** | 2.302878598 | 3.3977553 | 10 | 4 | EnergyNegMin |
| **BD without ADHD** | 0.906906907 | 1.761463413 | 10 | 1 | EnergyNegMin |
|  |  |  |  |  |  |
| **ADHD without BD** | 0.106643357 | 5.297207862 | 20 | 6 | EnergyMin |
| **BD with ADHD** | -0.907657658 | 4.502623756 | 20 | 4 | EnergyMin |
| **Other Diagnoses** | -2.133917397 | 5.13339243 | 20 | 6 | EnergyMin |
| **BD without ADHD** | -1.171171171 | 3.963403081 | 20 | 4 | EnergyMin |
|  |  |  |  |  |  |
| **ADHD without BD** | 5.248251748 | 4.336290119 | 20 | 8 | EnergyRange |
| **BD with ADHD** | 4.490990991 | 3.72441277 | 20 | 6 | EnergyRange |
| **Other Diagnoses** | 4.858573217 | 3.681393093 | 20 | 7 | EnergyRange |
| **BD without ADHD** | 4.432432432 | 3.351734729 | 17 | 5 | EnergyRange |
|  |  |  |  |  |  |
| **ADHD without BD** | 2.480769231 | 3.100982405 | 10 | 4 | AngerPosMax |
| **BD with ADHD** | 2.065315315 | 3.104320828 | 10 | 3 | AngerPosMax |
| **Other Diagnoses** | 2.13767209 | 3.164823907 | 10 | 3 | AngerPosMax |
| **BD without ADHD** | 2.465465465 | 2.91798167 | 10 | 5 | AngerPosMax |
