# Supplementary material for: Differentiating Pediatric Bipolar Disorder, Attention-Deficit/Hyperactivity Disorder, and Other Psychopathologies Using Self-Reported Mood and Energy Data and Actigraphy Findings: Correlation and Machine Learning–Based Prediction of Mood Severity
Source: JMIR Ment Health. 2025 Dec 4;12:e78163. doi: 10.2196/78163 (PMC12677876; doi:10.2196/78163)
Supplement: Multimedia Appendix 5 [file mental-v12-e78163-s005.docx]

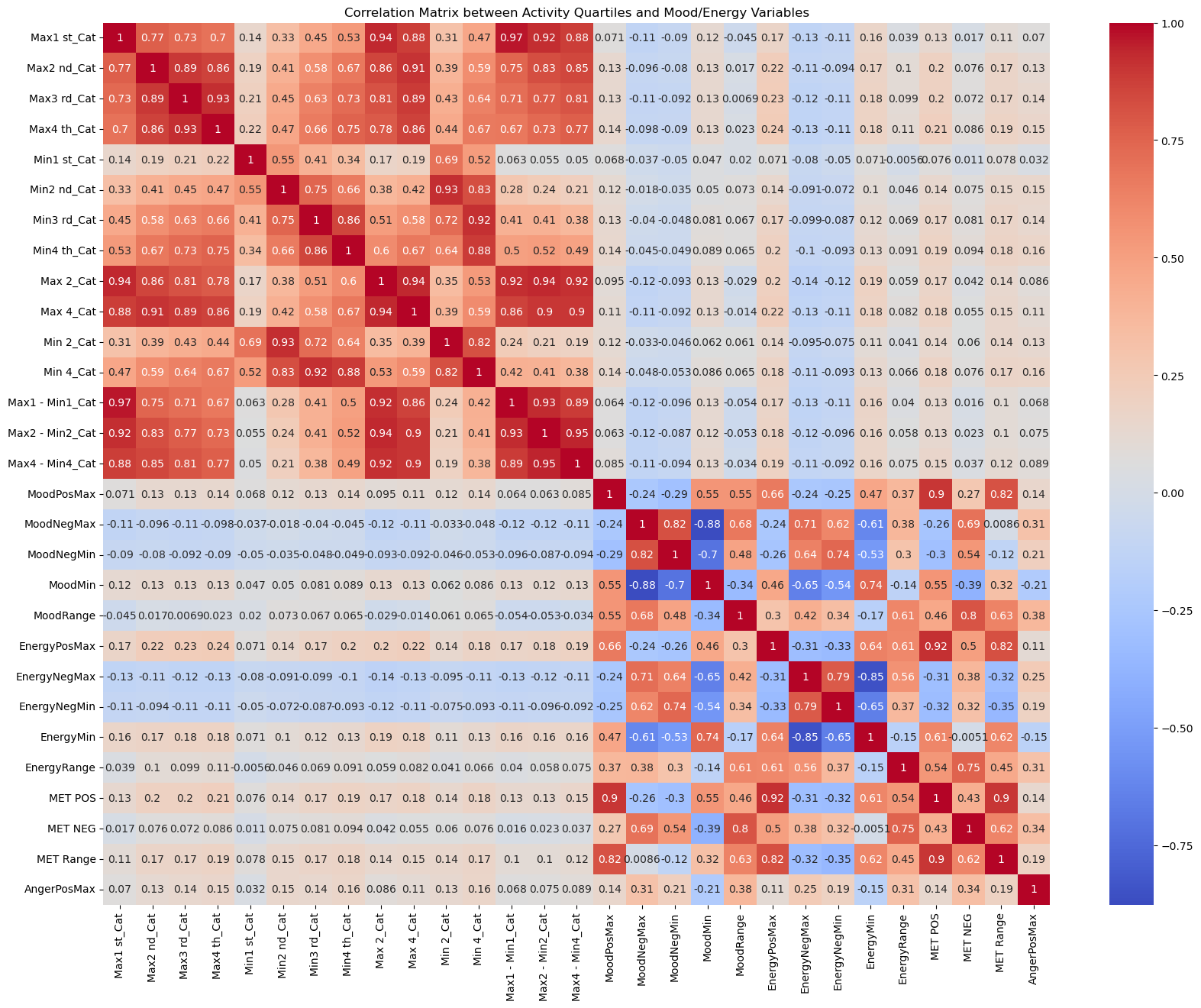


**Multimedia Appendix 5.** Correlation matrix between actigraphy-based activity quartiles and self-reported mood and energy variables.

This heatmap illustrates Pearson correlation coefficients between categorized activity quartiles (Max1–Max4, Min1–Min4) and mood/energy states measured by the Mood & Energy Thermometer (MET). Strong positive correlations were observed among different activity quartiles, while mood and energy variables showed distinct clustering patterns. Mood-related variables (e.g., MoodPosMax, MoodNegMax) were highly intercorrelated, as were energy-related variables (e.g., EnergyPosMax, EnergyNeg Max). However, correlations between activity levels and mood/energy variables were generally weak to moderate, highlighting the complexity of predicting subjective states from actigraphy-derived features
